# Development of an Optimized LC-MS Method for the Detection of Specialized Pro-Resolving Mediators in Biological Samples

**DOI:** 10.3389/fphar.2019.00169

**Published:** 2019-03-07

**Authors:** Laura Kutzner, Katharina M. Rund, Annika I. Ostermann, Nicole M. Hartung, Jean-Marie Galano, Laurence Balas, Thierry Durand, Michael S. Balzer, Sascha David, Nils Helge Schebb

**Affiliations:** ^1^Chair of Food Chemistry, Faculty of Mathematics and Natural Sciences, University of Wuppertal, Wuppertal, Germany; ^2^Institut des Biomolécules Max Mousseron (IBMM), UMR 5247 CNRS, ENSCM, Université de Montpellier, Montpellier, France; ^3^Division of Nephrology and Hypertension, Department of Medicine, Hannover Medical School, Hannover, Germany

**Keywords:** oxylipin, specialized pro-resolving mediators, inflammation, resolution, LC-MS

## Abstract

The cardioprotective and anti-inflammatory effects of long chain omega-3 polyunsaturated fatty acids (n3 PUFA) are believed to be partly mediated by their oxygenated metabolites (oxylipins). In the last two decades interest in a novel group of autacoids termed specialized pro-resolving mediators (SPMs) increased. These are actively involved in the resolution of inflammation. SPMs are multiple hydroxylated fatty acids including resolvins, maresins, and protectins derived from the n3 PUFA eicosapentaenoic acid (EPA) and docosahexaenoic acid (DHA) as well as lipoxins derived from arachidonic acid (ARA). In the present paper, we developed an LC-MS/MS method for a comprehensive set of 18 SPMs derived from ARA, EPA, and DHA and integrated it into our targeted metabolomics platform. Quantification was based on external calibration utilizing five deuterated internal standards in combination with a second internal standard for quality assessment of sample preparation in each sample. The tandem mass spectrometric parameters were carefully optimized for sensitive and specific detection. The influence of source parameters of the used AB Sciex 6500 QTRAP instrument as well as electronic parameters and the selection of transitions are discussed. The method was validated/characterized based on the criteria listed in the European Medicines Agency (EMA) guideline on bioanalytical method validation and method performance is demonstrated regarding recovery of internal standards (between 78 ± 4% and 87 ± 3% from 500 μL of human serum) as well as extraction efficacy of SPMs in spiked plasma (intra-day accuracy within ±20 and ±15% at 0.1 and 0.3 nM in plasma, respectively). Based on the lower limit of quantification of 0.02–0.2 nM, corresponding to 0.18–2.7 pg on column, SPMs were generally not detectable/quantifiable in plasma and serum supporting that circulating levels of SPMs are very low, i.e., <0.1 nM in healthy subjects. Following septic shock or peritonitis, SPMs could be quantified in the samples of several patients. However, in these studies with a small number of patients no clear correlation with severity of inflammation could be observed.

## Introduction

Inflammation is a defensive mechanism of the organism to respond to invading microorganisms or tissue injury. In an attempt to destroy pathogens and restore normal tissue function, inflammatory mediators, such as vasoactive amines and peptides, cytokines, chemokines, and lipid mediators are produced (Medzhitov, 2008). For example, lipid mediators derived from arachidonic acid (ARA), e.g., prostaglandin E2 (PGE_2_) and leukotriene B4 (LTB_4_) are released that act vasodilative (Higgs, 1986) and trigger the recruitment of neutrophils to the site of inflammation (Haribabu et al., 2000). This process results in a state of acute inflammation, which ideally leads to the elimination of the infectious agent and is self-limited (Serhan et al., 2015). In the past decades it was shown that the resolution of inflammation is an active process based on the production of pro-resolving mediators that inhibit neutrophil influx and stimulate monocytes and macrophages in order to remove apoptotic neutrophils and cell debris (Medzhitov, 2008; Serhan and Petasis, 2011).

**Figure 1:**
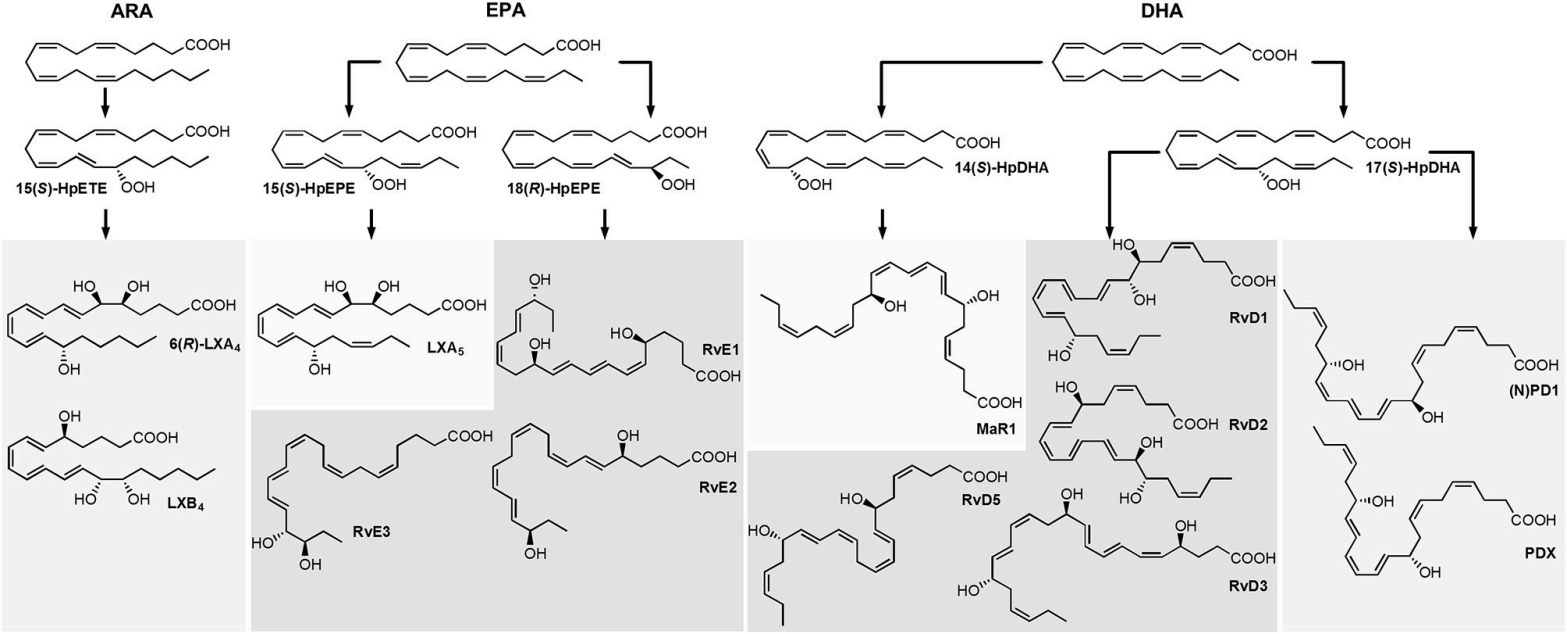
Structure and suggested formation routes of SPMs including ARA derived 4-series lipoxins, EPA derived 5-series lipoxin and E-series resolvins as well as DHA derived maresins, D-series resolvins and protectins.

Host defense and inflammation may be harmful to the organism if it fails to resolve the inflammation and return to homeostasis, and the resulting chronic inflammation is a leading cause of diseases (Serhan and Petasis, 2011; Calder, 2015). Resolution of inflammation is introduced by a lipid mediator class switching characterized by a shift from the predominantly pro-inflammatory mediators such as leukotrienes that amplify acute inflammation to the mostly anti-inflammatory pro-resolving lipoxins (LX) (Levy et al., 2001). Moreover, multiple hydroxylated fatty acids derived from the long-chain omega-3 polyunsaturated fatty acids (n3 PUFA) eicosapentaenoic acid (EPA) and docosahexaenoic acid (DHA) including resolvins (E- and D-series Rv), protectins and maresins (MaR) have been described that exert inflammation resolving properties (Figure 1) (Serhan, 2014; Bennett and Gilroy, 2016). These classes of bioactive molecules are enzymatically formed involving lipoxygenase (LOX), cyclooxygenase (COX) and may include cytochrome P450 (CYP) pathways and were termed specialized pro-resolving mediators (SPMs). As SPM production requires several conversion steps by enzymes which are not expressed in a single cell type, it thus requires the interplay of different cell types during the resolution of inflammation (Serhan et al., 2014). The anti-inflammatory activity of SPMs was demonstrated in *in vitro* and *in vivo* models of different inflammatory diseases and the widely appreciated health benefits associated with the intake of long-chain n3 PUFA might partly be based on the enhanced production of SPMs (Calder, 2015). For the formation of SPMs the time course has to be considered, as highest levels of SPMs are not observed during the initiation of inflammation but in the resolution phase (Serhan and Petasis, 2011; Werz et al., 2018).

Among the firstly recognized lipid mediators involved in the resolution of inflammation are ARA derived trihydroxy eicosatetraenoic acids that are formed in sequential lipoxygenations catalyzed by different LOX enzymes during cell-cell interactions and therefore referred to as lipoxins (LX) (Serhan et al., 1984; Serhan, 2005). Different routes of LX biosynthesis have been described: A double lipoxygenation of ARA catalyzed by 15-LOX and leukocyte 5-LOX leads to the formation of an epoxy-intermediate, which is enzymatically hydrolyzed to form both, 5(*S*),6(*R*),15(*S*)-trihydroxy eicosatetraenoic acid (6(*R*)-LXA_4_) or 5(*S*),14(*R*),15(*S*)-trihydroxy eicosatetraenoic acid (LXB_4_) (Figure 1) (Serhan, 2005). The other route of formation involves the 5-LOX initiated synthesis of LTA_4_ in human neutrophils and its subsequent lipoxygenation by platelet-type 12-LOX during neutrophil-platelet interactions (Serhan, 2005). While LOX catalyzed LX formation leads to 15(*S*)-LX, 15(*R*)-LX are formed by aspirin acetylated COX-2 (Claria and Serhan, 1995; Serhan, 2005). Resolvins (Rv) are formed during the resolution phase of acute inflammation partly by cell-cell interactions from the n3 PUFA EPA and DHA and are therefore categorized into E-series and D-series Rv, respectively (Figure 1) (Hong et al., 2003). E-series Rv are formed from 18(*R*)-hydro(pero)xy eicosapentaenoic acid (18(*R*)-H(p)EPE), a hydroxylation product of EPA. The route of formation of 18(*R*)-H(p)ETE is unclear and it may be catalyzed by acetylated COX-2 in the presence of aspirin (Serhan et al., 2000), by CYP (Arita et al., 2005) or autoxidation (Ostermann et al., 2015). Subsequent 5-lipoxygenation of 18(*R*)-H(p)EPE leads to the formation of both, 5(*S*),12(*R*),18(*R*)-trihydroxy eicosapentaenoic acid (RvE1) via enzymatic hydrolysis of an epoxy-containing intermediate or to 5(*S*),18(*R*)-dihydroxy-eicosapentaenoic acid (RvE2) (Tjonahen et al., 2006; Serhan and Petasis, 2011). However, also the formation of 18(*S*)-H(p)EPE by acetylated COX-2 and subsequent conversion to 18(*S*)-RvE1 and 18(*S*)-RvE2 was observed (Oh et al., 2011). Another pathway involves the action of 12/15-LOX on 18(*R*)- or 18(*S*)-H(p)EPE, leading to the formation of pro-resolving 17(*R*),18(*R*)-dihydroxy eicosapentaenoic acid (18(*R*)-RvE3) and 17(*R*),18(*S*)-dihydroxy eicosapentaenoic acid (18(*S*)-RvE3), respectively (Isobe et al., 2012, 2013).

D-series Rv are formed in two iterative lipoxygenation steps (Figure 1): 17(*S*)-hydro(pero)xy-docosahexaenoic acid (17(*S*)-H(p)DHA) is formed by 15-lipoxygenation from DHA and serves as substrate for 5-LOX in a second lipoxygenation step at the C-7 or C-4. Thereby, dihydroxylated RvD5 and trihydroxylated RvD1 and RvD2 are formed (C-7) as well as dihydroxylated RvD6 and trihydroxylated RvD3 and RvD4 (C-4) (Hong et al., 2003; Serhan and Petasis, 2011). A second class of pro-resolving mediators derived from DHA are dihydroxylated (neuro)protectins ((N)PD), e.g. 10(*R*),17(*S*)-dihydroxy-docosahexaenoic acid ((N)PD1) formed from 17(*S*)-H(p)DHA via an epoxide intermediate and subsequent enzymatic hydrolysis (Hong et al., 2003; Serhan et al., 2006; Balas et al., 2014). The 12-lipoxygenation product 14(*S*)-H(p)DHA serves as precursor for a third class of DHA derived SPMs that are synthesized by macrophages and involved in resolution of inflammation and therefore termed maresins (MaR) (Serhan et al., 2009). The proposed formation scheme includes—similar to (N)PD1—a single lipoxygenation step and formation of 7(*R*),14(*S*)-dihydroxy docosahexaenoic acid (MaR1) via an epoxide containing intermediate (Serhan et al., 2009).

Because SPM formation involves multiple enzymatic transformations and cell-cell interactions, concentrations of SPMs compared to their mono-hydroxylated precursors are low (Mas et al., 2012) and bioactivity of these potent mediators is reported for the picomolar to lower nanomolar range (Serhan, 2017). Hence, analysis of SPMs requires powerful selective and sensitive methodologies. Methodological approaches used for SPM detection include gas chromatography, which was applied e.g., for the characterization of LX (Brezinski and Serhan, 1991). Enzyme linked immunoassays can be used for the detection of single compounds (Chiang et al., 1998; Kirkby et al., 2013), though their specificity might be limited with respect to a large number of possible regio- and stereoisomers formed. Nowadays, methods used for identification and quantification of SPMs and other oxylipins are mostly based on reversed phase liquid chromatography (RP-LC) (Masoodi et al., 2008; Mas et al., 2012; Le Faouder et al., 2013; Colas et al., 2014; Jónasdóttir et al., 2015; Skarke et al., 2015), chiral LC (Oh et al., 2011; Homann et al., 2014; Toewe et al., 2018), or both (Massey and Nicolaou, 2013; Barden et al., 2015) hyphenated via electrospray ionization (ESI) to tandem mass spectrometric (MS/MS) detection. However, despite application of state-of-the-art LC-MS/MS based methodology, SPM detection in biological samples remains challenging. For example, results regarding the detection of SPMs in plasma from healthy individuals and correlation between n3 PUFA supplementation and plasma SPM levels are conflicting and the presence of SPMs in this matrix has been questioned (Murphy, 2015). Whereas the biosynthesis of SPMs in healthy individuals might be limited, increased SPM formation is expected in inflammatory diseases or in response to inflammatory stimuli. However, this could not be supported by Skarke et al. and no alteration of plasma SPM levels in response to bacterial lipopolysaccharide (LPS) during the inflammatory or resolution phase could be observed in healthy individuals (Skarke et al., 2015). In contrast, SPMs were detected in plasma from patients suffering severe sepsis at levels from ~1–500 pM (Dalli et al., 2017). Overall, it remains to be elucidated whether SPMs circulate in blood of healthy individuals and which endogenously formed SPMs are relevant in inflammation (Murphy, 2015; Skarke et al., 2015).

In order to enable these studies the most sensitive and accurate quantification in biological samples is required. Therefore, in the present paper we developed an LC-MS/MS method using one of the most sensitive MS instruments commercially available. A focus was set on the optimization of instrumental parameters, internal standard (IS) recovery, precision and accuracy for a comprehensive set of ARA, EPA, and DHA derived SPMs. Our LC-MS/MS method allows the simultaneous quantification of SPMs with other enzymatically and autoxidatively formed oxylipins. Method validation was performed oriented at the guideline by the European Medicines Agency (EMA) on bioanalytical method validation. Finally, the method was applied on clinically relevant human samples from patients with and without septic shock or peritonitis.

## Materials and Methods

### Chemicals

Authentic standard substances of SPMs were purchased from Cayman Chemicals (local distributor: Biomol, Hamburg, Germany), i.e., resolvins (Rv) RvE1, RvD1, 17(*R*)-RvD1, RvD2, RvD3, RvD5, maresins (MaR) MaR1 and 7(*S*)-MaR1, protectin PDX as well as lipoxins (LX) LXA_5_, 6(*R*)-LXA_4_, 15(*R*)-LXA_4_, 6(*S*)-LXA_4_, and LXB_4_ as well as deuterated IS including ^2^H_5_-RvD1, ^2^H_5_-RvD2, ^2^H_5_-LXA_4_, ^2^H_4_-LTB_4_, and ^2^H_4_-9,10-DiHOME. Additionally, Rv 18(*R*)-RvE2, 18(*R*)-RvE3 and 18(*S*)-RvE3, which were a kind gift of the lab of Makoto Arita (RIKEN Center for Integrative Medical Sciences, Japan) were synthesized as described (Ogawa et al., 2009; Isobe et al., 2012, 2013). (Neuro)protectin (N)PD1 was synthesized as follows: The (N)PD1-methyl ester was synthesized for its C10-epimer as described (Dayaker et al., 2014) replacing the (*S*)-1,2,4-butanetriol by its (*R*)-enantiomer as starting material for the introduction of the *E,E*-iododiene. Methyl ester-(N)PD1 was than hydrolyzed with 1 M LiOH in MeOH/H_2_O (1/1) followed by acidification with McIlvains buffer (pH 5) producing (N)PD1 as a colorless oil in 97% yield. Acetonitrile (ACN), LC-MS grade methanol (MeOH) and acetic acid were obtained from Fisher Scientific (Schwerte, Germany). HPLC grade *n*-hexane and disodium hydrogen phosphate dihydrate were purchased from Carl Roth (Karlsruhe, Germany). All other chemicals were purchased from Sigma Aldrich (Schnelldorf, Germany). Pure water was generated by a GenPure UF/UV Ultrapure water system from TKA Wasseraufbereitungssysteme GmbH (Niederelbert, Germany). For human plasma generation human whole blood was collected into EDTA monovettes (S-Monovette K3E, 02.1066.001, Sarstedt, Nümbrecht, Germany), centrifuged (15 min, 4°C, 1,200 × g) and plasma was pooled (five healthy volunteers, aged 25–38 years). For human serum generation human whole blood was collected into monovettes (S-Monovette with clotting activator, 02.10063, Sarstedt, Nümbrecht, Germany), incubated for 30 min at room temperature, centrifuged (10 min, 4°C, 2,500 × g) and serum was pooled (three healthy female subjects, aged 26–27 years). Plasma and serum were immediately stored at −80°C until analysis.

### Mass Spectrometric Optimization

Mass spectrometric detection was performed on a 6500 QTRAP instrument (AB Sciex, Darmstadt, Germany) coupled to a 1290 Infinity LC System (Agilent, Waldbronn, Germany). Analyses were carried out in negative electrospray ionization (ESI(-)) mode. The influence of source parameters [electrode protrusion, probe x- and y-axis position, source temperature, nebulizer gas (GS1) and auxiliary (drying) gas (GS2)] was assessed in flow injection analysis (FIA) mode injecting 5 μL of a standard solution (100 nM) at a flow rate of 300 μL min^−1^ (ca. 50% Solvent B, see below). For the Ion Drive Turbo V source (AB Sciex, Darmstadt, Germany) the ESI probe can be arranged along the y-axis (0 to 13 mm, with 13 mm representing the closest position relative to the orifice) and optimization ranges were chosen from 0.0 to 5.0 mm in steps of 0.5 mm. Along the x-axis (0 to 10 mm, with 5 mm as center position relative to the orifice) the ESI probe was adjusted from 2.5 to 7.5 mm in steps of 0.5 mm. Additionally, the protrusion of the electrode was adjusted with typical values ranging from <0.5 to 2 mm. The source temperature was ranged between 300 and 550°C (with constant GS2 60 psi), the pressure of the auxiliary (drying) gas (GS2) was ranged from 40 to 70 psi (with constant temperature 475°C) and the nebulizer gas (GS1) between 30 and 70 psi.

For MS optimization collision induced dissociation (CID) fragment spectra were monitored (100 nM standard solution) applying a CE range between −16 and −30 V depending on the substance. Two to three of the most intense and specific fragments were selected and individually optimized regarding the adjustment of electronic parameters including declustering potential (DP), collision energy (CE), collision cell exit potential (CXP) as well as collision activated dissociation (CAD) gas pressure. Optimization ranges for these parameters were chosen as follows: DP from −20 to −100 V in steps of 10 V, CE from −13 to −31 (to −39 for RvD2 *m/z* 175.0) in steps of 2 V and re-optimized in steps of 1 V, CXP from −4 to −18 V in steps of 2 V. Influence of CAD gas was assessed for representative compounds in low (6 psi), medium (9 psi) and high (15 psi) mode for different CEs.

### LC-MS/MS Method

Chromatographic separation was performed on a Zorbax Eclipse Plus C18 reversed phase column (2.1 × 150 mm, particle size 1.8 μm, pore size 9.5 nm; Agilent, Waldbronn, Germany) using a binary gradient. Solvent A was 0.1% acetic acid mixed with 5% solvent B and solvent B was ACN/MeOH/acetic acid (800/150/1, *v/v/v*). The flow rate was set to 0.3 mL min^−1^ and the linear gradient was as follows: 21% B at 0 min, 21% B at 1.0 min, 26% B at 1.5 min, 51% B at 10 min, 66% B at 19 min, 98% B at 25.1 min, 98% B at 27.6 min, 21% B at 27.7 min and 21% B at 31.5 min. For MS detection the 6500 QTRAP mass spectrometer (AB Sciex, Darmstadt, Germany) was operated in negative electrospray ionization (ESI(-)) mode. Nitrogen was used as curtain gas and CAD gas (nitrogen generator IMT-PN1450 PAN, INMATEC, Herrsching, Germany). Zero air was used as nebulizer (GS1) and drying gas (GS2) generated with an air compressor (SL-S 5.5, Renner, Güglingen, Germany) and zero air generator (UHP-300-ZA-S-E, Parker, Kaarst, Germany). In the optimized method, the probe position was 0.250 cm along the vertical (y-) axis and 0.550 cm along the horizontal (x-) axis, electrode protrusion was between 1 and 1.5 mm, ion spray voltage was −4500 V, curtain gas (N_2_) was kept at 35 psi, nebulizer gas (GS1) and drying gas (GS2) were adjusted to 60 psi each and source temperature was 475°C. Detection was carried out in scheduled selected reaction monitoring (SRM) mode (detection window 90 s, cycle time 0.4 s) with the CAD gas set to 15 psi and individually optimized electronic parameters for each SPM (Table 1). In addition to the SPMs the method covered the quantitative detection of 175 enzymatically and chemically formed oxylipins as described (Rund et al., 2017).

**Table 1:** Optimized parameters of the LC-ESI(-)-MS/MS method for the quantification of SPMs.

**Analyte**	**Mass transition**			**MS parameters**				**Internal standard**	**Retention time^**a**^**	**FWHM^**b**^**	**LOD**^**c**^		**Calibration range**^**d**^		
													**LLOQ (5 μL)^**e**^**	**LLOQ (10 μL)^**e**^**	**ULOQ^**f**^**
		**Q1**	**Q3**	**DP**	**EP**	**CE**	**CXP**		**[min]**	**[s]**	**[nM]**	**[pg on column]**	**[nM]**		
5(*S*),6(*R*),15(*S*) LXA_4_	(1)	351.2	115.2	−40	−10	−20	−8	^2^H_5_-LXA_4_	10.19	3.5	0.18	0.31	0.25	0.18	500
	(2)	351.2	235.0	−40	−10	−20	−12				0.75	1.3	1.0	0.50	500
	(3)	351.2	217.1	−40	−10	−27	−12				2.0	3.5	5.0	2.0	500
5(*S*),6(*R*),15(*R*) ${\text{LXA}}_{4}^{\text{h}}$	(1)	351.2	115.0	−40	−10	−20	−8	^2^H_5_-LXA_4_	10.26	3.7	0.18	0.31	0.25	0.18	500
	(2)	351.2	235.1	−40	−10	−20	−12				0.75	1.3	1.0	0.50	500
	(3)	351.2	217.1	−40	−10	−27	−12				2.0	3.5	5.0	2.0	500
5(*S*),6(*S*),15(*S*) LXA_4_	(1)	351.2	115.1	−40	−10	−20	−8	^2^H_5_-LXA_4_	10.57	3.5	0.18	0.31	0.25	0.18	500
	(2)	351.2	235.2	−40	−10	−20	−12				0.50	0.88	1.0	0.50	500
	(3)	351.2	217.1	−40	−10	−27	−12				2.0	3.5	5.0	2.0	500
LXB_4_	(1)	351.2	221.0	−40	−10	−22	−13	^2^H_5_-LXA_4_	9.19	3.5	0.50	0.88	1.0	0.50	500
	(2)	351.2	233.1	−40	−10	−22	−13				0.50	0.88	1.0	0.50	500
	(3)	351.2	251.0	−40	−10	−19	−13				1.5	2.6	5.0	2.0	500
LXA_5_	(1)	349.1	114.9	−40	−10	−19	−5	^2^H_5_-LXA_4_	8.77	3.2	0.10	0.18	0.25	0.18	500
	(2)	349.1	215.0	−40	−10	−25	−13				0.50	0.88	1.0	0.50	500
	(3)	349.1	233.1	−40	−10	−19	−11				0.75	1.3	1.0	0.75	500
RvE1	(1)	349.3	195.0	−50	−10	−23	−10	^2^H_5_-RvD2	6.19	3.5	0.38	0.66	0.5	0.25	500
	(2)	349.3	161.0	−50	−10	−25	−8				0.75	1.3	1.0	0.75	500
	(3)	349.3	205.0	−50	−10	−22	−10				1.0	1.8	2.0	1.0	500
RvE2	(1)	333.2	253.3	−60	−10	−20	−9	^2^H_4_-9,10-DiHOME	11.37	3.8	1.0	1.7	2.0	1.0	100
	(2)	333.2	159.2	−60	−10	−25	−10				1.0	1.7	2.0	1.0	100
	(3)	333.2	199.1	−60	−10	−23	−10				1.0	1.7	2.0	1.0	100
18(*S*) RvE3	(1)	333.2	201.3	−60	−10	−20	−9	^2^H_4_-9,10-DiHOME	12.75	3.8	0.50	0.84	1.0	0.50	100
	(2)	333.2	245.3	−60	−10	−18	−9				0.50	0.84	1.0	0.50	100
18(*R*) RvE3	(1)	333.2	201.3	−60	−10	−20	−9	^2^H_4_-9,10-DiHOME	13.42	4.0	0.25	0.42	0.50	0.25	100
	(2)	333.2	245.3	−60	−10	−18	−9				0.25	0.42	0.50	0.25	100
RvD1^g^	(1)	375.3	141.0	−40	−10	−20	−8	^2^H_5_-RvD1	10.32	3.6	–	–	0.25	0.18	500
	(2)	375.3	215.0	−40	−10	−27	−13				–	–	0.25	0.18	500
	(3)	375.3	233.3	−40	−10	−20	−12				–	–	0.50	0.25	500
17(*R*) RvD1^h^	(1)	375.3	140.9	−40	−10	−20	−8	^2^H_5_-RvD1	10.41	3.6	0.38	0.71	0.75	0.38	500
	(2)	375.3	215.1	−40	−10	−27	−13				0.50	0.94	0.75	0.38	500
	(3)	375.3	233.0	−40	−10	−20	−12				0.75	1.4	1.5	0.75	500
RvD2	(1)	375.3	175.0	−50	−10	−31	−12	^2^H_5_-RvD2	9.52	3.8	0.38	0.71	0.75	0.38	500
	(2)	375.3	141.0	−50	−10	−23	−8				0.75	1.4	1.0	0.50	500
	(3)	375.3	277.0	−50	−10	−19	−14				0.75	1.4	1.5	0.75	500
RvD3	(1)	375.3	147.0	−60	−10	−27	−10	^2^H_5_-RvD2	9.20	3.6	0.18	0.33	0.38	0.18	500
	(2)	375.3	137.0	−60	−10	−27	−8				1.0	1.9	2.0	1.0	500
	(3)	375.3	181.0	−60	−10	−23	−10				1.5	2.8	5.0	2.0	500
RvD5	(1)	359.1	199.1	−40	−10	−23	−10	^2^H_4_-LTB_4_	13.80	4.4	0.38	0.68	0.75	0.38	500
	(2)	359.1	141.0	−40	−10	−20	−8				1.0	1.8	2.0	1.0	500
	(3)	359.1	261.0	−40	−10	−19	−14				2.0	3.6	5.0	2.0	500
MaR1	(1)	359.1	250.2	−50	−10	−21	−12	^2^H_4_-LTB_4_	13.81	4.2	1.5	2.7	2.0	1.0	500
	(2)	359.1	177.0	−50	−10	−23	−10				2.0	3.6	5.0	2.0	500
	(3)	359.1	221.0	−50	−10	−20	−8				2.0	3.6	5.0	2.0	500
7(*S*)-MaR1	(1)	359.1	250.1	−50	−10	−21	−12	^2^H_4_-LTB_4_	13.25	4.2	0.25	0.45	0.50	0.25	500
	(2)	359.1	177.0	−50	−10	−23	−10				1.5	2.7	2.0	1.0	500
	(3)	359.1	221.0	−50	−10	−20	−8				1.5	2.7	5.0	2.0	500
(N)PD1	(1)	359.0	153.0	−50	−10	−21	−8	^2^H_4_-9,10-DiHOME	13.48	4.1	0.25	0.45	0.50	0.25	500
	(2)	359.0	206.0	−50	−10	−21	−12				0.18	0.32	0.38	0.18	500
PDX	(1)	359.1	153.1	−50	−10	−22	−8	^2^H_4_-9,10-DiHOME	13.71	4.1	0.18	0.32	0.25	0.18	500
	(2)	359.1	206.1	−50	−10	−22	−12				0.10	0.18	0.25	0.18	500
^2^H_5_-RvD2		380.2	175.0	−55	−10	−31	−10		9.47	3.5					
^2^H_5_-LXA_4_		356.3	222.2	−55	−10	−25	−13		10.13	3.6					
^2^H_5_-RvD1		380.3	141.0	−50	−10	−19	−8		10.26	3.6					
^2^H_4_-LTB_4_		339.2	197.2	−65	−10	−23	−9		13.97	4.3					
^2^H_4_-9,10-DiHOME		317.2	203.4	−80	−10	−29	−8		15.11	4.6					

*Shown are all SPMs covered by the method with their mass transitions for quantification in scheduled SRM mode, electronical MS parameters [declustering potential (DP), entrance potential (EP), collision energy (CE), collision cell exit potential (CXP)], the assigned internal standards (IS), retention time (RT), peak width, limit of detection (LOD) and the calibration range [lower limit of quantification (LLOQ), upper limit of quantification (ULOQ)]*.

a *Relative standard deviation for RT within one batch was ≤ 0.10 % (±0.01 min)*.

b *Full peak width at half maximum (FWHM) was determined as mean width of standards, concentration LLOQ-500 nM*.

c *LOD was set to the lowest concentration yielding a signal to noise ratio ≥3*.

d *Calibration was performed as weighted regression using 1/x^2^ weighting*.

e *LLOQ was set to the lowest calibration standard injected yielding a signal to noise ratio ≥ 5 and an accuracy within ± 20%*.

f *ULOQ concentration does not represent the end of the dynamic range, but is the highest calibration standard injected*.

g *No determination of LOD due to impurity of IS ^2^ H_5_-RvD1; LLOQ was set to lowest concentration yielding an S/N ≥ 5 and an accuracy within ±20%*.

h *Compounds 17(R)-RvD1 and 15(R)-LXA_4_ were not included in the calibration mixture and quantification is based on the calibration curves of their isomers RvD1 and 5(S),6(R),15(S)-LXA_4_, respectively*.

### Method Characterization

Method characterization and validation was carried out in terms of sensitivity, linearity, intraday precision and accuracy, oriented at the guideline of the European Medicine Agency (EMA) for bioanalytical method development (EMEA/CHMP/EWP/ Rev. 1 Corr. 2., 2011). Calibration standards covering a concentration range from LLOQ up to 500 nM (100 nM for RvE2, 18(*R*)- and 18(*S*)-RvE3) of SPMs were measured and linearity was assessed by plotting the peak area ratio (analyte/IS) against the analyte concentration (linear least square regression, weighting 1/x^2^). Accuracy was within ±15% of the nominal concentration (except ±20% for LLOQ). Intraday accuracy and precision were assessed in plasma spiked with a subset of SPMs at four different concentration levels (0.1, 0.3, 1, and 3 nM in plasma) and additionally in serum at one concentration level (3 nM in serum). SPMs were spiked into plasma/serum samples directly at the beginning of sample preparation and unspiked plasma and serum was prepared alongside. Accuracy was determined by comparison of the determined concentration to the concentration in the spiking standard solution. Precision was calculated as relative standard deviation (*n* = 4).

Extraction efficacy of the deuterated internal standards was determined by calculation of the recovery rates utilizing an internal standard 2 added at the end of sample preparation. For evaluation of ion suppression effects by the matrix IS was spiked into serum at the beginning of sample preparation and into the serum extract at the end of sample preparation (post SPE).

### Sample Preparation

SPMs were extracted from plasma or serum samples and effluents from peritoneal dialysis (PD) using solid phase extraction (SPE) (Rund et al., 2017). In the first step a mixture of 20 deuterated IS (20 nM each, including ^2^H_5_-RvD1, ^2^H_5_-RvD2, ^2^H_5_-LXA_4_, ^2^H_4_-LTB_4_, and ^2^H_4_-9,10-DiHOME), antioxidant mixture (0.2 mg/mL BHT, 100 μM indomethacin, 100 μM soluble epoxide hydrolase inhibitor *trans*-4-[4-(3-adamantan-1-yl-ureido)-cyclohexyloxy]-benzoic acid (*t*-AUCB) in MeOH) were added to 500 μL of plasma/serum or 1,200 μL of PD exudates. Then 1,400 μL ice-cold MeOH (3,360 μL for PD exudates) were added for protein precipitation (at least 30 min at −80°C). Following centrifugation, the supernatant was evaporated under a gentle nitrogen stream to <50% MeOH, diluted with 0.1 M disodium hydrogen phosphate buffer (pH 5.5) and loaded onto the preconditioned SPE column (Bond Elut Certify II, 200 mg, 3 mL; Agilent, Waldbronn, Germany). Oxylipins were eluted with ethyl acetate/*n*-hexane (75/25, *v/v*) containing 1% acetic acid. After evaporation to dryness in a vacuum concentrator (30°C, 1 mbar, ca. 60 min; Christ, Osterode, Germany) sample extracts were reconstituted in 50 μL MeOH containing 40 nM 1-(1-(ethylsulfonyl)piperidin-4-yl)-3-(4-(trifluoromethoxy)phenyl)urea as IS 2. Injection volume was 5 μL; for samples with low SPM content a second (10 μL) injection was used for SPM quantification.

### Clinical Samples

#### Peritoneal Dialysis Patient Samples

Serum and peritoneal dialysate effluent samples from peritoneal dialysis (PD) patients from the Hannover Medical School PD outpatient clinic were obtained after written informed consent according to the declaration of Helsinki, and local ethics board approval (MHH 2014/6617). Patients were treated exclusively with biocompatible PD fluids. Dialysate samples (1-2 L) with an intra-abdominal presence ≥2 h were drained via PD catheter from the abdomen of patients with peritonitis (*n* = 4–5) and from clinically stable control patients (*n* = 4–5), respectively, and immediately frozen at −80°C until further analysis. After coagulation in the fridge serum was centrifuged within 4 h after sampling (10 min, 2,300 × g) and frozen at −80°C until further analysis. In accordance with the current International Society for Peritoneal Dialysis recommendations on prevention and treatment of PD-related peritonitis (Li et al., 2016) a diagnosis of peritonitis was made when at least 2 of the following were present: (1) clinical features consistent with peritonitis, i.e., abdominal pain and/or cloudy dialysis effluent; (2) dialysis effluent white cell count >100/μL (after a dwell time of at least 2 h), with >50% polymorphonuclear; and (3) positive dialysis effluent culture.

#### Septic Shock Patient Samples

Plasma samples were obtained from patients with septic shock per SEPSIS-3 definition (Singer et al., 2016) at the Hannover Medical School ICU or healthy controls after written informed consent according to the declaration of Helsinki and approved by the Hannover Medical School Ethical Committee (2786-2015). Included were 18 patients with early septic shock (<12 h) and high need for high doses of norepinephrine (>0.4 μg/kg/min) that were neither pregnant, aged <18 years nor had an end-stage chronic disease. All patients were part of the recently published EXCHANGE trial (Knaup et al., 2018). Blood was drawn within 12 h after diagnosis and plasma was centrifuged within <6 h after sampling (10 min, 3,500 × g) and frozen at −80°C until further analysis.

## Results and Discussion

In order to enable sensitive and selective detection of SPMs electronic MS parameter were carefully optimized for each compound and the impact of source parameters on sensitivity was thoroughly assessed.

### Optimization of Mass Spectrometric Detection

The influence of source parameters (probe position, source gases, source temperature) on sensitivity was assessed. Three SPMs, i.e., RvE1, RvD2 and RvD5 were chosen representing the structure of di- and trihydroxy fatty acids and a broad elution window and thus different compositions of the mobile phase during elution and evaporation with retention times of 6.19 min (RvE1, 40% solvent B), 9.52 min (RvD2, 50% solvent B) and 13.80 min (RvD5, 57% solvent B). The protrusion of the electrode was adjusted from <0.5 mm to 2 mm and showed only a little effect on the sensitivity of SPM detection (Supplementary Information). Even though signal intensity was higher (ca. 10%) with small protrusion, the signal was more unstable and noisier compared to a higher protrusion. Therefore, 1–1.5 mm was found to be optimal, consistent with manufacturer recommendation. Moving the ESI probe closer toward the orifice along the y-axis (from 0 to 5 mm), which is the closest recommended position for typical LC flow rates of 200–1,000 μL min^−1^, yielded a 36% higher signal for RvE1 (40% solvent B) and only 17% for the later eluting RvD5 (57% solvent B). The probe position along the y-axis was set to 2.5 mm resulting in 9–20% lower signal compared to the position at 5 mm (Supplementary Information). However, during the analysis of biological specimen a position of the probe close to the orifice leads to a transfer of neutral compounds and thus a rapid contamination of the MS. Unexpectedly, moving the ESI probe farther right along the x-axis (5–7.5 mm) gave higher signal intensity of 7–10% compared to directly before the orifice (5 mm), whereas movement to the left side (5–2.5 mm) resulted in 17–27% lower signal intensity. Hence, an off-center x-position of the ESI probe of 5.5 mm was chosen (Supplementary Information).

Overall it can be concluded that the signal intensity can be further improved by maximal 15% (protrusion), 20% (y-axis), and 10% (x-axis) compared to the chosen values and has therefore only little influence on the performance of the MS for the detection of SPMs (Supplementary Information). This can be explained by the wide heating region and a large spray cone compared to a relatively small orifice in the ion source. Thus, it can be concluded that using a medium value of the recommended ranges for the probe position seems to be sufficient as default position for the detection of SPMs and other oxylipins.

**Figure 2:**
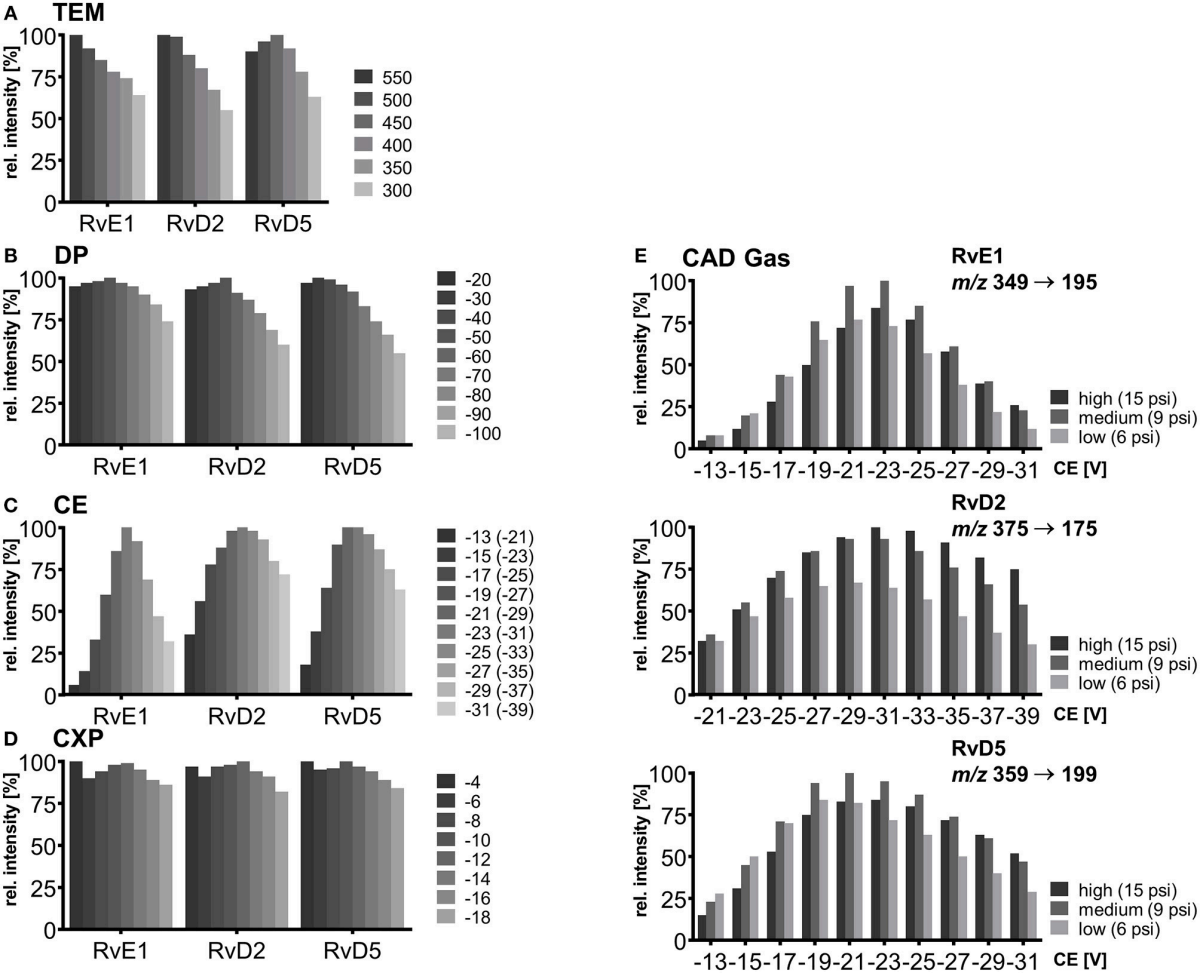
Mass spectrometric optimization of electronic parameters as well as collision gas and source temperature in SRM mode for compounds RvE1, RvD2, and RvD5. Shown is the influence of parameters on signal intensity within a range around the optimum value. **(A)** Source temperature (TEM) between 300 and 550°C, **(B)** declustering potential (DP) between −20 and −100 V **(C)** collision energy (CE) in steps of 2 V from −13 to −31 V for RvE1 (*m/z* 349 → 195) and RvD5 (*m/z* 359 → 199), CE from −21 to −39 V for RvD2 (*m/z* 375 → 175) with collision activated dissociation (CAD) gas set to high (15 psi), **(D)** collision cell exit potential (CXP) in steps of 2 V from −4 to −18 V, **(E)** collision energy (CE) in steps of 2 V from −13 to −31 V for RvE1 (*m/z* 349 → 195) and RvD5 (*m/z* 359 → 199), CE from −21 to −39 for RvD2 (*m/z* 375 → 175) with collision activated dissociation (CAD) gas set to high (15 psi), medium (9 psi) and low (6 psi).

In the next step, the influence on signal intensity of nebulizer gas (GS1) as well as auxiliary gas (GS2) from the two heated jets and its temperature was assessed. With optimized source gases a signal gain of 16–23% (GS1) and 6–12% (GS2) can be achieved (Supplementary Information) and 60 psi was selected for both gases. The temperature was found to be a critical parameter and was optimized in a range from 300 to 550°C. Higher GS2 temperature led to higher intensities for RvE1 and RvD2 (36 and 45% signal gain with 550°C compared to 300°C). In contrast, for later eluting RvD5 maximal intensity was observed at TEM 450°C (Figure 2A) indicating a thermal degradation at higher temperature. This is not only a solvent evaporation effect, because for PGE_2_ (RT 8.99 min) a decreasing signal intensity was also found above 400°C. Thus, the temperature has to be carefully optimized, since higher temperature improves desolvatization of stable compounds but is disadvantageous for thermo-labile compounds, particularly those eluting with high percentage of organic solvent leading to higher thermal stress due to faster solvent evaporation. We selected a source temperature of 475°C as compromise allowing the detection of all SPMs as well as other oxylipins. This is consistent with other methods using 400 to 580°C for the detection of SPMs on the same instrument type (Jónasdóttir et al., 2015; Vlasakov et al., 2016).

Careful optimization of the electronic parameters declustering potential (DP), collision energy (CE) and collision cell exit potential (CXP) was carried out for each individual compound and the influence of the collision activated dissociation (CAD) gas pressure was determined: For DP the impact on signal intensity was minimal (<10%) within a range of −20 to −60 V, while higher DPs led to a declining signal (Figure 2B). The DP (similar to the potential termed nozzle-skimmer voltage, cone voltage in instruments from other companies) which is applied to the orifice plate allows the dissociation of ion clusters. High DP leads to in-source fragmentation due to collision with gas molecules taking place at the relatively low vacuum in the transfer region (Gabelica and De Pauw, 2005). Because of the large orifice of the instrument, which is a major difference compared to older models of the type of instrument, only little influence on the signal results for oxylipins within a DP between −40 and −60 V. Therefore, a standard DP of −60 V could be selected for all oxylipins, even though we used the optimal parameters for the SPMs (Table 1).

As expected, CE led to massive differences in signal intensities of 64–94% from highest to lowest within the tested range (−13 to −31 V; −21 to −39 V for RvD2) emphasizing the significant impact of CE on occurrence and extent of collision induced fragmentation (CID) and thus sensitivity in SRM mode (Figure 2C). Fine tuning was performed for each of the CEs in steps of 1 V and variation of CEs ±1 V around the optimum caused a signal decline of maximal 5% indicating that—though a critical parameter—CE optimization in smaller steps than 2 V is not required for our instrument. For most SPMs CEs from −19 to −23 V were optimal for all fragments, in some cases such as for RvD2 (*m/z* 375.3 → 175.0) higher CEs (−25 to −31 V) were required to provide sufficient energy for fragmentation in order to ensure sensitive detection. For different adjustments of CAD gas pressure the trend of CEs and optimal CE were mostly similar (high, 15 psi; medium, 9 psi; low, 6 psi). CAD gas set to “low” gave overall lowest intensities at optimal CE, while highest intensities were observed for CAD gas set to “medium” or “high” depending on the fragment ion (Figure 2E). This may be caused by increasing probability for ions to undergo CID with higher CAD gas pressure (Sleno and Volmer, 2004). On the other hand higher potential at lower gas pressure might lead to more intense collisions because of higher kinetic energy of the ions. Optimal CAD gas pressure gives 16–33% higher signal intensities compared to the lowest pressure. In our method, CAD gas is set to high resulting in lower intensities for e.g., RvE1 (*m/z* 349.3 → 195.0) or RvD5 (*m/z* 359.1 → 199.1) and better sensitivity for RvD2 (*m/z* 375.3 → 175.0). Optimal collision cell exit potential (CXP) was between −8 and −14 V for all compounds and ±2 V around the optimum caused a signal decline of maximal 6% (Figure 2D). Therefore, despite an optimal CXP was chosen, a standard default value of −10 V seems to be suitable for SPMs and other oxylipins.

**Figure 3:**
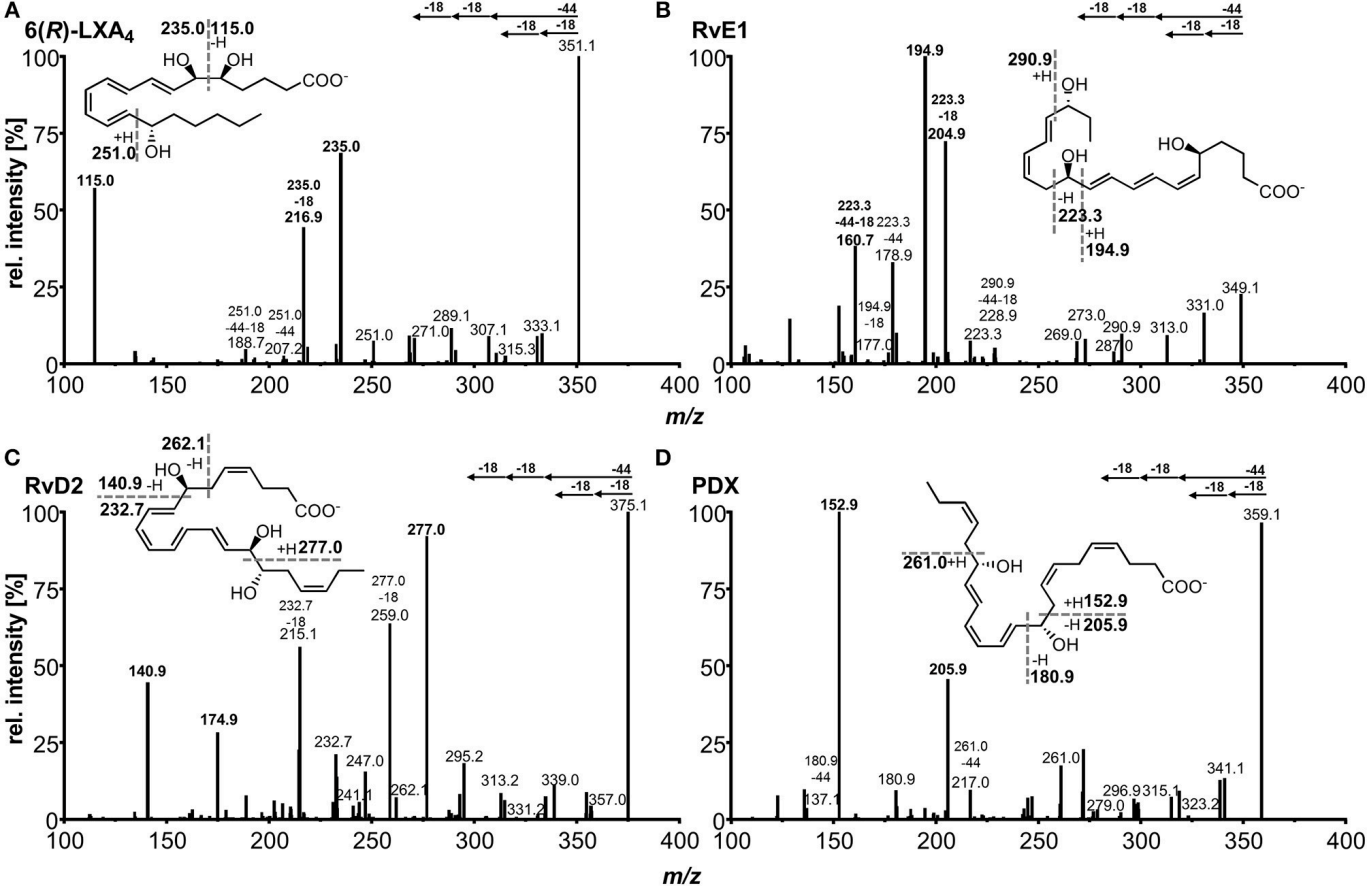
Collision induced dissociation (CID) product ion spectra of representative SPMs comprising **(A)** ARA derived lipoxin 5(*S*),6(*R*),15(*S*)-LXA_4_, **(B)** EPA derived E-series resolvin RvE1, **(C)** DHA derived D-series resolvin RvD2, **(D)** DHA derived protectin PDX. The dashed lines in the structures depict suggested sites of fragmentation leading to specific transitions.

For each compound two or three specific transitions were chosen, as exemplarily shown in Figures 3A–D, to ensure both selective and sensitive detection and thus quantification alongside with identification of SPMs in biological sample material. For most of the compounds the transition with highest sensitivity, i.e., best signal-to-noise ratio was selected as primary transition, whereas alternative transitions were comparable (e.g., RvD2, PDX) or less sensitive (e.g., RvD3, 6(*R*)-LXA_4_) (Table 1). For method characterization, quantification was carried out on all transitions and concentrations determined with different transitions were compared e.g., in order to evaluate matrix interferences and support compound identity. For all compounds α-cleavage ions referring to a cleavage of the carbon chain in α-position of the hydroxyl group with a double bond in β- or γ-position (α-hydroxy-β/γ-ene fragmentation mechanism) with or without an additional loss of H_2_O/CO_2_ were used for quantification. Their formation has been described for SPMs and other oxylipins earlier (Murphy et al., 2005; Hong et al., 2007; Lu et al., 2007). For example, the most sensitive transition selected for RvE1 (*m/z* 349.3 → 195.0) is based on α-hydroxy-β-ene rearrangement, the alternative transitions (*m/z* 349.3 → 205.0, *m/z* 349.3 → 161.0) are formed in an α-hydroxy-γ-ene rearrangement with elimination of H_2_O (*m/z* 205.0) and of H_2_O/CO_2_ (*m/z* 161.0) (Figure 3B) (Lu et al., 2007). For RvD2 the most sensitive fragment (*m/z* 375.3 → 175.0) is unlikely to be formed by an α-cleavage and may be formed by a γ-cleavage toward the hydroxyl group or another mechanism (Figure 3C). However, as this fragment is the most sensitive with our instrument and is also used by other groups for RvD2 (Barden et al., 2014; Homann et al., 2014; Toewe et al., 2018), it was chosen as primary transition. These backbone fragments [“chain-cut ions” (Hong et al., 2007)] are specific allowing to discriminate between regioisomers, whereas fragments referred to as “peripheral-cut ions” (Hong et al., 2007) that result from the unspecific loss of water (hydroxyl group) and/or carbon dioxide (carboxylic group) are not selective and do not allow to draw conclusions on the position of the hydroxyl groups being essential for the selective detection of e.g., RvD5, PD1, and MaR1 (DHA-derived dihydroxy-FA, Q1 mass: *m/z* 359.1) and other isobaric autoxidation products which could be formed from PUFA. Similar fragments were observed for the other SPMs and specific transitions chosen are consistent with literature (Hassan and Gronert, 2009; Mas et al., 2012; Le Faouder et al., 2013; Massey and Nicolaou, 2013; Colas et al., 2014; Homann et al., 2014; Jónasdóttir et al., 2015; Skarke et al., 2015; Vlasakov et al., 2016). Overall, the selection of appropriate transitions is a crucial step for the detection of SPMs and other oxylipins. Due to multiple hydroxyl groups most SPMs give rise to intense ions originating from a cleavage within the molecular backbone allowing specific detection.

### Chromatographic Separation

**Figure 4:**
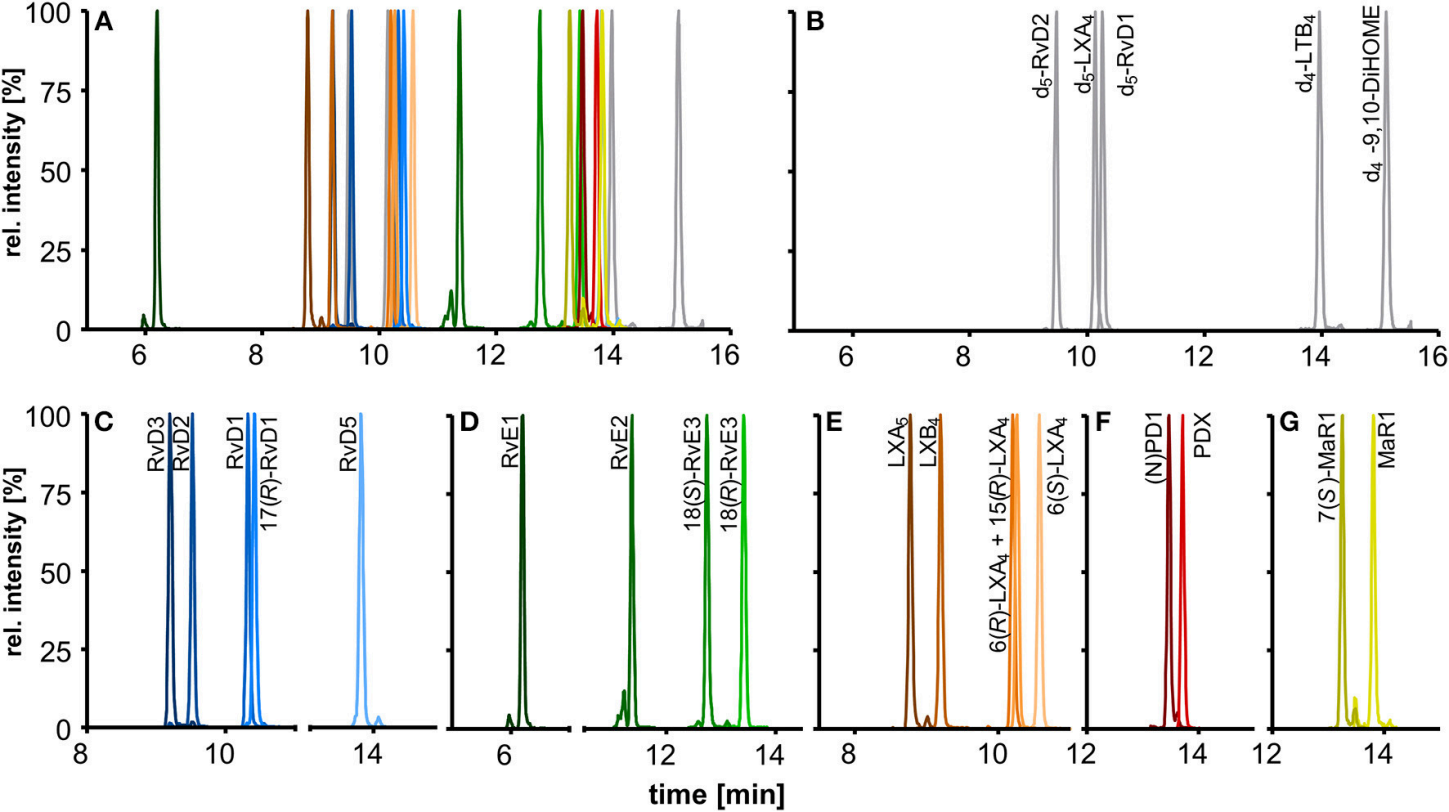
Chromatographic separation of **(A)** 18 SPMs (100 nM each) and **(B)** 5 deuterated internal standards (20 nM) covered by the method including **(C)** DHA derived D-series resolvins, **(D)** EPA derived E-series resolvins, **(E)** EPA and ARA derived lipoxins, DHA derived **(F)** protectins, and **(G)** maresins. Separation was carried out on an RP-18 column (2.1 × 150 mm, particle size 1.8 μm, pore size 9.5 nm) with a H_2_O/MeOH/ACN/HAc gradient.

The chromatographic separation was carried out on a state-of-the-art C18 reversed phase column filled with sub-2 μm particles and optimized gradient. In addition to the optimized detection described here the chromatographic separation enables the simultaneous analysis of 175 enzymatically and autoxidatively formed lipid mediators within 31.5 min (Rund et al., 2017). The optimized method covers a total of 18 SPMs that elute in the first part of the chromatogram within 10 min and allows the chromatographic resolution of most of the SPMs yielding narrow peaks with a peak width at half maximum (FWHM) of 3–4 s (Figures 4A–G).

The chromatographic separation of SPMs is crucial due to the large number of stereo- and regioisomers regarding position and configuration of the hydroxyl group (*R, S*) bearing carbons and the conjugated double bonds (*E, Z*) that exhibit identical fragmentation patterns and similar chromatographic behavior (Hansen et al., 2016).

Our method allows sufficient chromatographic resolution (R) of the critical separation pairs, namely stereoisomers MaR1 and 7(*S*)-MaR1 (*R* = 4.7) and the protectins (N)PD1 and PDX (*R* = 2.0) (Figures 4F,G). The two aspirin-triggered isomers 17(*R*)-RvD1 and 15(*R*)-LXA_4_ are not baseline separated from 17(*S*)-RvD1 (*R* = 0.9) and 6(*R*)-LXA_4_ (*R* = 0.6), respectively (Figures 4C,E). With this performance the method is better or comparable to that reported by other groups (Sun et al., 2007; Mas et al., 2012), while baseline separation can be achieved by using chiral stationary phases (Massey and Nicolaou, 2013; Homann et al., 2014; Lehmann et al., 2015). Based on the incomplete separation, 17(*R*)-RvD1 and 15(*R*)-LXA_4_ were not included in the calibration mixture and quantification was based on calibration curves of 17(*S*)-RvD1 and 6(*R*)-LXA_4_, respectively.

For RvD5 (RT 13.80 min), MaR1 (RT 13.81 min) and PDX (RT 13.71 min) with an *m/z* [M-H]^−^ of 359.1 the choice of specific (and alternative) transitions is crucial to differentiate between these compounds and to allow the specific quantification. The fragments *m/z* 359.1 → 250.2 (MaR1(1)) and *m/z* 359.1 → 153.1 (PDX(1)) are the most intense and specific fragments for MaR1 and PDX, respectively. However, for RvD5 (*m/z* 359.1 → 199.1) both MaR1 (0.6%) and PDX (3%) show a signal on this transition. To ensure a reliable quantification of RvD5 in presence of high PDX concentrations a second transition (*m/z* 359.1 → 141.0, RvD5(2)) was therefore included as alternative fragment.

### Sensitivity

**Figure 5:**
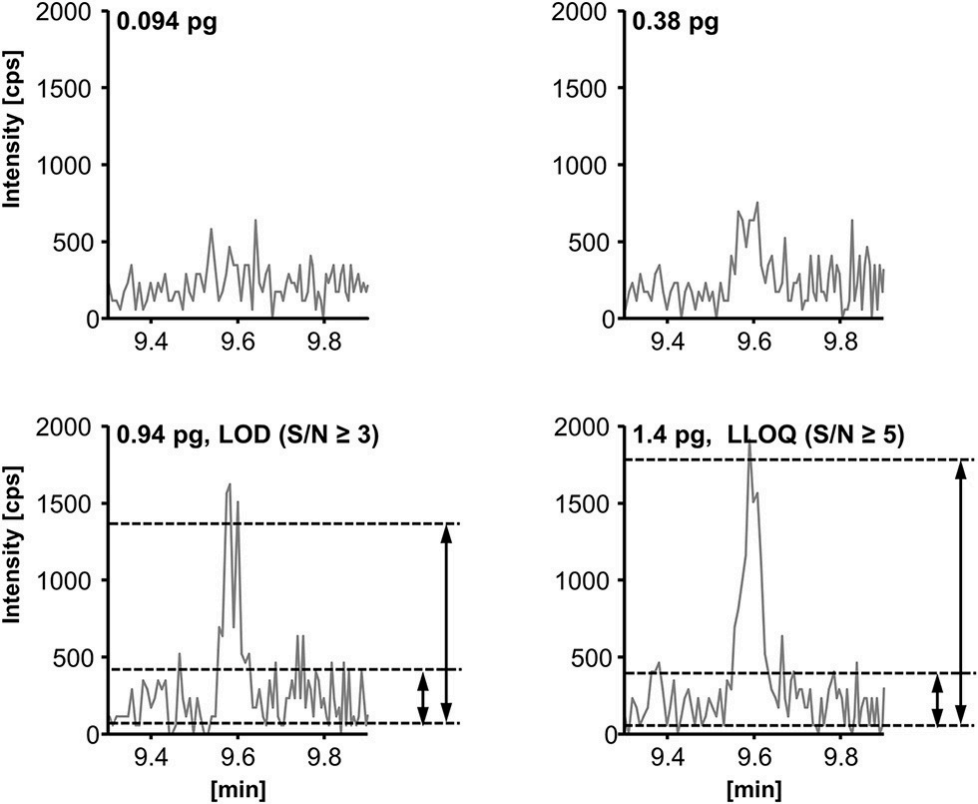
Determination of the limit of detection (LOD) and the lower limit of quantification (LLOQ) exemplarily shown for resolvin D2 (RvD2, *m/z* 375.3 → 175.0). LOD is defined as peak-to-peak signal-to-noise-ratio (S/N) ≥ 3 and LLOQ as (S/N) ≥ 5 and an accuracy within ±20% of the nominal concentration.

The limit of detection (LOD) and lower limit of quantification (LLOQ) were determined according to the EMA guideline for bioanalytical methods (EMEA/CHMP/EWP/ Rev. 1 Corr. 2., 2011). The LOD was set to the lowest (calibration) standard injected yielding a signal-to-noise-ratio (S/N) ≥ 3; the LLOQ was set to the lowest calibration standard yielding an S/N ≥ 5 and an accuracy within ±20% of the nominal concentration. The S/N (peak-to-peak) was determined manually as exemplarily shown for RvD2 (Figure 5, for LOD and LLOQ of exemplary SPMs see Supplementary Information). As listed in Table 1, for our method the LOD was between 0.1 and 1.5 nM (0.18–2.7 pg on column) for the most sensitive transition, whereas for alternative transitions similarly low or higher LODs were determined. Despite different instrumentation, comparable or slightly higher detection limits are reported in literature, e.g., 3 pg on column (Mas et al., 2012), 1.3–4.9 pg on column (Le Faouder et al., 2013), 0.10–5.2 pg on column in plasma sample (Skarke et al., 2015) of which all used an S/N of at least 3 as criterion. It should be noted that LODs of as low as 0.02 pg are reported for the same instrument as used in our lab (Colas et al., 2014). However, the use of different criteria for LOD determination might explain this huge difference of 1–2 orders of magnitude. The LLOQ ranged from 0.25 to 2.0 nM (corresponding to 0.025–0.2 nM in plasma/serum) for the quantifier. Slightly better i.e., lower LLOQs can be achieved with higher injection volume (10 μL: LLOQ from 0.18 to 1.0 nM (0.018–0.1 nM in plasma/serum), Table 1). Further increasing the injection volume resulted in an inacceptable peak shape due to reconstitution of the sample extract in pure organic solvent (Rund et al., 2017). Reconstitution of the sample extract in a 1:1 methanol/water mixture allows for higher injection volumes (up to 20 μL) with acceptable peak shape for the analytes. However, less polar oxylipins were not sufficiently dissolved leading to unacceptable low recoveries of e.g., epoxy-FA but also monohydroxy-FA (Supplementary Information). Therefore, it would not be possible to accurately quantify SPM precursors such as 17-HDHA or CYP-derived oxylipins, which may be used as indicators for n3-PUFA supplementation (Murphy, 2015), parallel to SPMs. Overall, it can be summarized that in our hands under optimized conditions the lowest concentration which can be quantified for SPMs and other oxylipins is about 1 nM in the injected solvent corresponding to about 1 pg on column.

### IS Recovery and Ion Suppression

**Figure 6:**
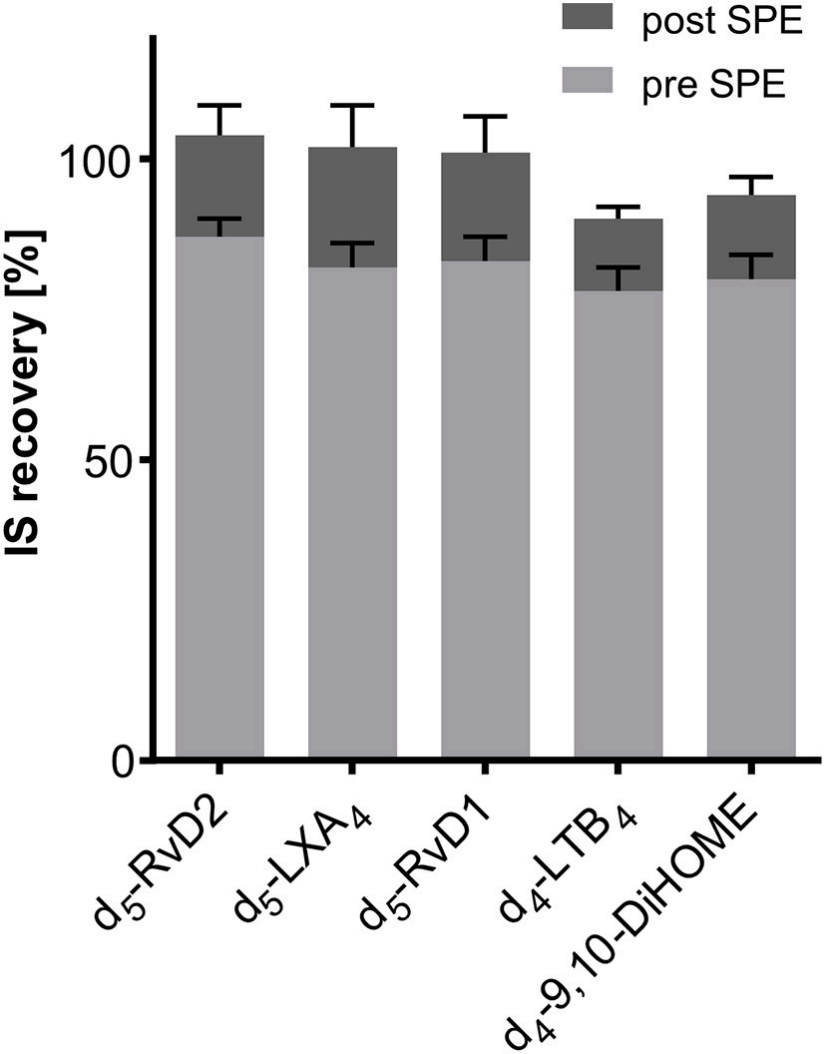
Recovery of deuterated internal standards (IS) used for quantification of SPMs in 500 μL serum. Recovery of IS 1 (added directly at the beginning of sample preparation) was determined utilizing 1-(1-(ethylsulfonyl)piperidin-4-yl)-3-(4-(trifluoromethoxy)phenyl)urea as IS 2 (added after sample preparation directly before measurement). Evaluation of ion suppression was based on IS recovery when IS was added directly before reconstitution of sample extract after SPE.

Recovery of internal standards (IS) used for SPM quantification was between 78 ± 4% (^2^H_4_-LTB_4_) and 87 ± 3% (^2^H_5_-RvD2) from 500 μL of human serum (*n* = 3, Figure 6). If the IS was added after the solid phase extraction step recovery rates were between 90 ± 2% (^2^H_4_-LTB_4_) and 105 ± 5% (^2^H_5_-RvD2) (Figure 6). From this it can be concluded that with the SPE procedure as established in our laboratory (Rund et al., 2017) IS are sufficiently well extracted from matrix (>75%) and matrix effects are efficiently reduced (maximal ±10%). It should be noted that IS for quantification of all other oxylipins covered by our method show good recoveries from matrix between 72 ± 3% (^2^H_8_-5-HETE) and 105 ± 6% (^2^H_11_-5(*R*,*S*)-5-F_2t_-IsoP) (Supplementary Information). Thus, it can be assumed that a method allowing a good recovery rate of both polar oxylipins such as prostanoids (e.g., PGE_2_) and less polar hydroxy-PUFA (e.g., 5-HETE) is also appropriate for the extraction of SPMs.

### Extraction Efficacy and Intraday Accuracy and Precision

In order to evaluate the accuracy and precision of the quantification of concentrations of SPMs in biological samples, human plasma (with SPM levels <LOD) was spiked at four concentration levels (0.1, 0.3, 1, and 3 nM in plasma) with a subset of SPMs. At 0.1 nM plasma concentration and 5 μL injection volume all of the spiked compounds were detected with at least two different specific transitions (Table 2). Accuracies were within ±20% of the nominal (added) concentration for the quantifier (except RvE1) and precisions were <20%. For some less sensitive alternative transitions 0.1 nM was below LLOQ leading to higher variation of the determined concentrations, as e.g., 82 ± 35% for RvD3 *m/z* 375.3 → 137.0 (Table 2). However, as lower LLOQs can be achieved with higher injection volume, better accuracies and precisions were obtained with an injection volume of 10 μL, e.g., 103 ± 15% for RvD3 *m/z* 375.3 → 137.0 (Table 2). For spiking levels from 0.3 to 3 nM in plasma determined concentrations using the quantifier were within ±15% compared to the added concentration and precision <15%. However, also quantification using alternative transitions resulted in acceptable accuracy (maximal ±21%) and precision (<16%) for concentrations >LLOQ. The only exception is RvE1, which was quantified with an accuracy of 68–81% for all spiking levels in plasma, most likely due to interferences by the plasma matrix, e.g., ion suppression, which was not observed in human serum (accuracy 108% for RvE1, Table 2). Matrix interference could also lead to the slightly higher determined concentrations for RvD5 (121–122%) with an injection volume of 10 μL (Table 2). In summary, all three chosen transitions were suitable for quantification of SPMs in human plasma and serum; however, for routine measurement the two most sensitive transitions (one quantifier and one qualifier ion) seem to be sufficient. In the unspiked plasma/serum of healthy individuals used for the spiking experiment no SPMs could be detected, i.e., they did not exceed the LOD. It should be noted that we found good recoveries in freshly spiked human plasma and serum samples, however, the SPMs could be degraded during storage of the samples. Though most oxylipins are stable during storage at −80°C (Jonasdottir et al., 2018) lower SPM levels have been reported in plasma which was stored for a longer period of time (Colas et al., 2014). It would be important to investigate the stability of naturally formed SPMs as well as spiked analytes in future studies because this could lead to the high concentration differences reported in biological samples. Regarding SPM levels in human plasma and/or serum reported concentrations differ considerably. For example, in baseline human plasma concentrations of RvD1 and RvE1 that lie within the working range of our method were reported, such as 0.0454 nM (RvD1) and 0.521 - 1.00 nM (RvE1) (Psychogios et al., 2011), 0.10–0.11 nM (RvD1) and 0.11–0.14 nM (RvE1) (Barden et al., 2014). However, also concentrations below our LLOQ were found in human plasma, e.g., 0.007 nM RvD1 (Colas et al., 2014) or were not detected at all (Skarke et al., 2015). In human serum SPM amounts were considerably higher compared to the plasma analyzed in the same study, probably due to formation during coagulation (Colas et al., 2014). Interestingly, in another study with plasma and serum from healthy volunteers after n3-PUFA supplementation comparable SPM levels were found in plasma and serum (Mas et al., 2012). Therefore, differences in sample generation, handling and storage may impact detectability and quantity of low levels of SPMs. In our study we could not detect SPMs in blank plasma and serum, while in spiked samples SPM levels as low as 0.1 nM could be detected. Thus, our study supports earlier reports that the circulating levels of SPMs in healthy individuals are very low, as described e.g., by Colas et al. (2014). In order to come to comparable results regarding the concentration of SPMs in biological samples all methods used should be validated based on internationally accepted guidelines. Moreover, direct comparison of results obtained by different, independent laboratories as e.g., performed by Norris et al. (2018) in form of round robin trials are required.

**Table 2:** Intraday accuracy (acc.) and precision (prec.) for the extraction of a subset of SPMs from human plasma (500 μL) and human serum (500 μL).

**Analyte**	**Mass transition**			**0.1 nM in plasma**^**a**^				**0.3 nM in plasma**^**a**^				**1.0 nM in plasma**		**3.0 nM in plasma**		**3.0 nM in serum**	
				**5** **μL**		**10** **μL**		**5** **μL**		**10** **μL**		**5** **μL**		**5** **μL**		**5** **μL**	
		**Q1**	**Q3**	**acc. [%]**	**prec. [%]**	**acc. [%]**	**prec. [%]**	**acc. [%]**	**prec. [%]**	**acc. [%]**	**prec. [%]**	**acc. [%]**	**prec. [%]**	**acc. [%]**	**prec. [%]**	**acc. [%]**	**prec. [%]**
6(*R*)-LXA_4_	(1)	351.2	115.2	94	14	93	5	100	10	92	4	98	2	99	4	97	3
	(2)	351.2	235.0	72	11	83	9	88	9	79	6	94	2	99	4	94	4
	(3)	351.2	217.1	<LOD		75	22	76	7	94	11	99	5	98	5	98	3
6(*S*)-LXA_4_	(1)	351.2	115.1	82	17	87	4	93	4	86	5	85	0	88	5	88	1
	(2)	351.2	235.2	71	22	73	8	86	5	83	4	87	2	88	2	87	5
	(3)	351.2	217.1	<LOD		102	11	95	5	87	2	85	8	87	5	87	2
LXB_4_	(1)	351.2	221.0	105	5	106	9	96	8	97	4	98	5	100	3	95	3
	(2)	351.2	233.1	109	14	119	10	103	8	97	7	96	3	100	4	92	2
	(3)	351.2	251.0	<LOD		<LOD		102	9	101	15	93	2	100	5	95	6
LXA_5_	(1)	349.1	114.9	114	7	119	7	110	5	114	4	111	4	112	3	102	4
	(2)	349.1	215.0	97	9	105	5	106	3	112	1	105	5	111	4	102	4
	(3)	349.1	233.1	86	9	100	7	93	4	109	7	106	5	113	4	103	6
RvE1	(1)	349.3	195.0	68	8	78	4	73	6	81	6	69	8	71	7	108	2
	(2)	349.3	161.0	81	17	73	15	74	11	79	6	72	11	68	5	105	6
	(3)	349.3	205.0	<LOD		72	7	62	12	79	7	73	5	69	6	108	5
RvD1	(1)	375.3	141.0	94	9	96	4	98	3	91	3	96	7	100	1	97	1
	(2)	375.3	215.0	94	8	93	4	91	7	93	4	93	5	98	4	98	4
	(3)	375.3	233.3	80	5	82	14	89	9	91	8	95	6	101	1	98	1
RvD2	(1)	375.3	175.0	101	8	98	3	96	5	104	4	98	7	100	4	105	3
	(2)	375.3	141.0	99	10	98	7	91	6	94	5	94	9	97	3	108	2
	(3)	375.3	277.0	105	13	93	5	105	2	98	5	98	9	96	2	105	3
RvD3	(1)	375.3	147.0	105	8	103	6	109	5	103	4	106	7	103	3	113	2
	(2)	375.3	137.0	82	35	103	15	102	9	103	11	106	10	105	4	115	4
	(3)	375.3	181.0	<LOD		128	11	101	17	105	6	112	14	106	4	116	6
RvD5	(1)	359.1	199.1	109	3	122	11	111	4	121	4	109	10	107	4	106	3
	(2)	359.1	141.0	111	6	110	12	107	16	116	8	102	11	104	6	107	5
	(3)	359.1	261.0	<LOD		<LOD		115	7	98	8	108	13	108	3	100	5
MaR1	(1)	359.1	250.2	110	19	119	14	109	10	107	4	105	8	104	5	105	5
	(2)	359.1	177.0	88	24	122	12	99	7	107	6	93	11	100	6	101	3
	(3)	359.1	221.0	<LOD		<LOD		110	11	113	5	92	11	104	2	104	7
7(*S*)-MaR1	(1)	359.1	250.1	97	6	94	12	99	4	102	2	91	9	96	4	103	2
	(2)	359.1	177.0	105	9	90	18	99	2	103	7	99	5	97	6	103	3
	(3)	359.1	221.0	<LOD		<LOD		110	13	108	3	95	11	97	4	104	3
(N)PD1	(1)	359.0	153.0	87	17	114	9	91	11	95	3	98	6	92	4	98	3
	(2)	359.0	206.0	95	8	99	15	109	8	96	6	98	7	89	3	105	5
PDX	(1)	359.1	153.1	96	5	106	3	98	2	99	4	94	6	93	5	102	3
	(2)	359.1	206.1	103	6	96	6	95	6	95	5	89	8	90	4	101	2

*SPMs were spiked into plasma at four concentration levels of 0.1, 0.3, 1.0, and 3.0 nM and additionally into serum at the highest concentration level (3.0 nM in serum). Accuracy was calculated based on the determined concentration in sample after sample preparation utilizing SPE extraction in comparison to the concentration in the spiking standard solution. Precision was expressed as relative standard deviation of the sample set (n = 4). For concentration levels 0.1 and 0.3 nM acc. and prec. for 5 and 10 μL injection volume are shown. Quantification was carried out for the quantifier (most sensitive transition) as well as for the alternative transitions if concentration was >LOD. No calculation of accuracy and precision if analyte was in ≥50% of the samples <LOD*.

a *concentrations <LLOQ and >LOD were quantified to calculate accuracy and precision*.

Ongoing work aims to address these questions.

### SPM Formation in Peritonitis

**Figure 7:**
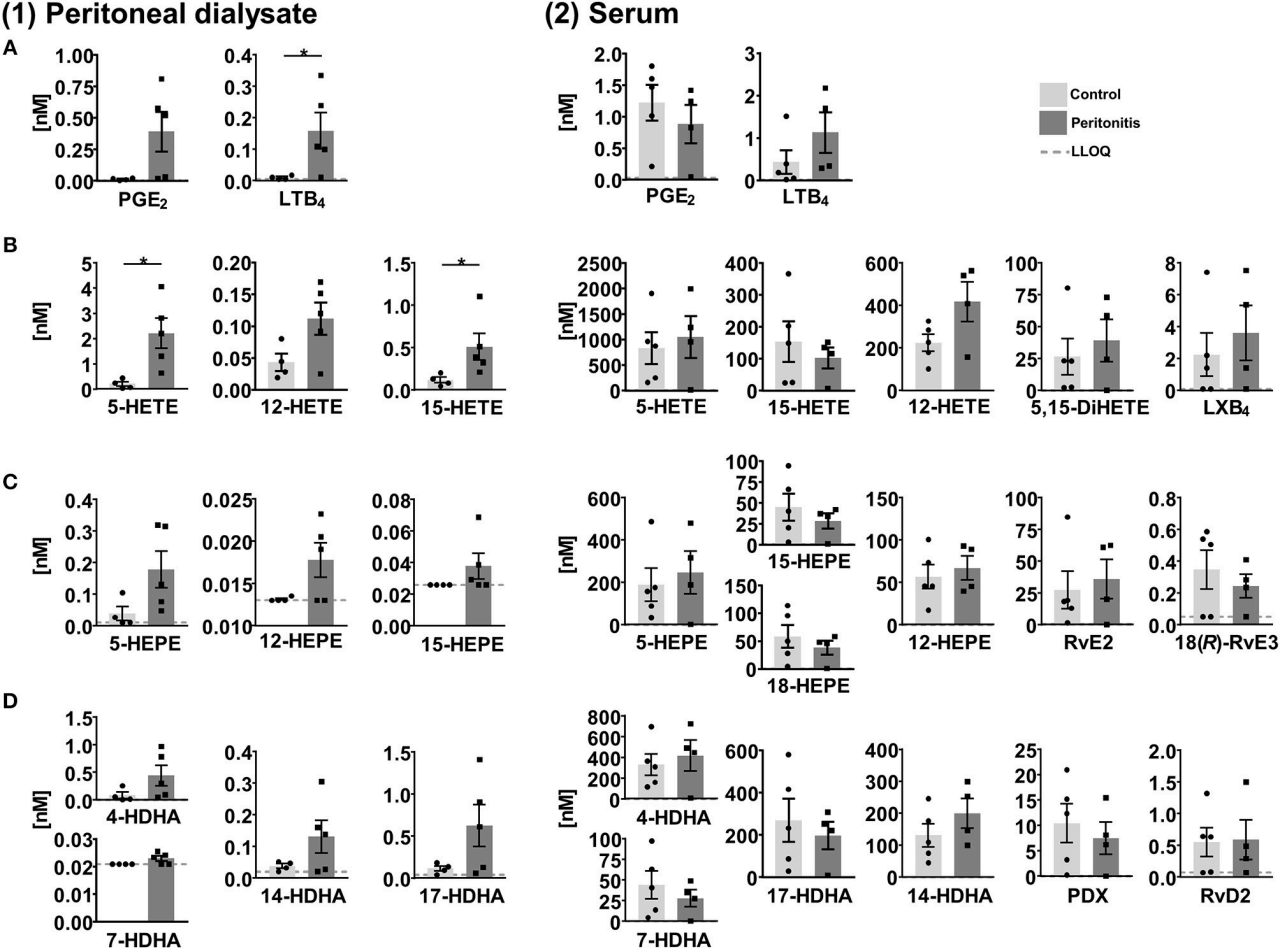
Concentration of selected lipid mediators measured in **(1)** peritoneal dialysate and **(2)** serum from patients with end stage renal disease treated with peritoneal dialysis (PD) with (peritonitis, *n* = 4–5) or without (control, *n* = 4–5) acute inflammation. Shown are concentrations in nM as individual values and mean ± SEM of **(A)** ARA derived pro-inflammatory lipid mediators, 5-, 12-, and 15-lipoxygenation products/SPM precursors as well as di- and tri-oxygenation products/SPMs derived from **(B)** ARA, **(C)** EPA, and **(D)** DHA. For concentrations <LLOQ, the LLOQ is given. Mean ± SEM are only calculated if >50% of the samples are >LLOQ. The LLOQ is indicated as dotted line. In dialysates of the control group one outlier was eliminated based on Grubb's test (α = 0.05). Statistically significant differences between control and peritonitis group are indicated by ^*^p < 0.05 calculated by Mann-Whitney *U* test.

SPMs and other oxylipins were quantified in peritoneal dialysate and serum samples, which were obtained from patients with end stage renal disease treated by peritoneal dialysis (PD) as renal replacement therapy (Ellam and Wilkie, 2015) with (peritonitis, *n* = 4–5) and without (control, *n* = 4–5) acute inflammation. In peritoneal effluents pro-inflammatory mediators PGE_2_ and LTB_4_ were elevated in the peritonitis group compared to the control group (Figure 7) and similar trends were observed for the 5-, 12-, and 15-LOX products. However, SPMs were detected only in a single sample and therefore not displayed in Figure 7. 15-lipoxygenation products were quantified only in low concentrations (≤1 nM) and SPM precursor 18-HEPE was <LLOQ in >50% of the samples. In the serum 12 SPMs could be quantified in >50% of the samples including di- and trihydroxylated ARA, EPA and DHA derived PUFA ranging from concentrations as low as 0.24±0.074 nM (18(*R*)-RvE3) to 36±15 nM (RvE2) in the peritonitis group (Figure 7, Supplementary Information). Overall, SPM concentrations as well as their precursors showed no significant difference between peritonitis and control group. A trend toward higher 5- and 12-lipoxygenation and lower 15-lipoxygenation products and 18-HEPE in peritonitis could be observed with high inter-individual variation, while for SPMs no consistent trend toward an elevation or reduction in peritonitis was observed (Figure 7). For SPMs that were detected in the PD patients' serum samples (e.g., RvE2, RvD2) a pro-resolving action in peritonitis was reported earlier, mainly observed as a reduction of PMN recruitment [summarized in Serhan (2010) and Recchiuti and Serhan (2012)]. However, these effects as well as the presence of SPMs in peritoneal lavages were mostly shown in zymosan induced murine peritonitis models and data on human clinical samples are scarce. Surprisingly, no detectable levels of SPMs were found in the dialysate of PD patients, despite being in direct contact to the inflamed tissue within a confined space. A reason could be the time point for sample collection, as SPM concentrations change during the inflammation/resolution process. In murine peritonitis models SPM formation was reduced after the initiation of inflammation (highest after 2–6 h, reduced/not detectable after 9–24 h) (Bannenberg et al., 2005; Fredman et al., 2012; Divanovic et al., 2013), with induction of a more severe, non-self-resolving inflammation (Chiang et al., 2012; Fredman et al., 2012) or were not detected (Dioszeghy et al., 2008; Spite et al., 2009). In clinical samples, the individual time-course and severity of the inflammation as well as the strong dilution of lipid mediators in the PD solution (1–2 L) or differences in sample collection and processing in the clinical daily routine can have an influence on lipid mediator levels and could explain SPM levels <LLOQ in the dialysate from peritonitis patients. The SPM pathway markers such as 17-HDHA were elevated in the peritonitis group compared to control group and could serve as indicator for potential SPM formation during peritonitis. In contrast, the overall similar levels in the serum of control and peritonitis patients of both pro- and anti-inflammatory lipid mediators might indicate a sustained systemic inflammation of end stage renal disease patients. Due to the reduction in renal clearance, poor biocompatibility of PD fluids and oxidative stress, chronic inflammation processes are enhanced in long-term PD patients (Lai and Leung, 2010; Velloso et al., 2014). Therefore, elevated levels of inflammation-associated lipid mediators, small sample size and high inter-individual variation might mask differences in SPM concentrations between control and peritonitis group.

### SPM Formation in Septic Shock

**Figure 8:**
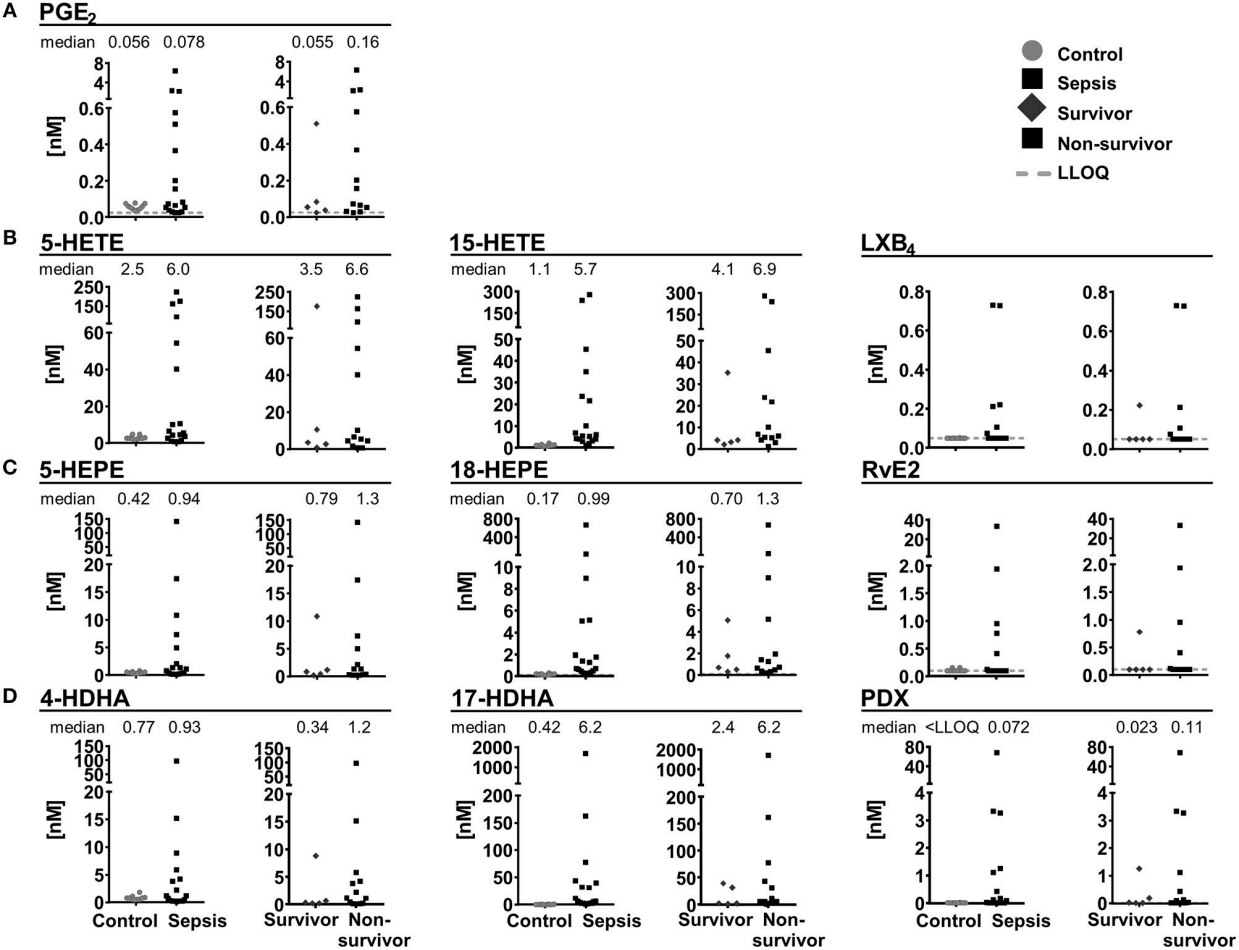
Concentration of selected lipid mediators measured in plasma from patients with (sepsis, *n* = 18) and without (control, *n* = 10) septic shock. Patients with septic shock are divided into survivors (>28 days, *n* = 5) and non-survivors (*n* = 13). Shown are concentrations in nM as individual values of **(A)** ARA derived PGE_2_, 5-lipoxygenation products and SPM pathway marker as well as SPMs derived from **(B)** ARA, **(C)** EPA, and **(D)** DHA. Median is given, if >50% of the samples are >LLOQ. The LLOQ is indicated as dotted line.

In plasma samples from patients with septic shock [*n* = 18, APACHE II score 41.5 (22–52)], severe clinical and humoral signs of inflammation [CRP 236 mg/L (68–422 mg/L)] and multi-organ failure, SPMs and other oxylipins were quantified and compared to plasma samples of healthy individuals serving as control (*n* = 10). In control samples, 4 SPMs (RvE2, LXB_4_, (N)PD1 and PDX) were detected and exceeded LLOQ only in 2 individuals (0.027–0.16 nM). In plasma from septic shock patients, 12 SPMs were quantified with large inter-individual variation ranging from <LLOQ (<0.018 nM) up to >20 nM (Figure 8). Most SPMs including LX, E-, and D-series Rv were quantified in less than half of the study population (1–6 individuals), whereas protectins (N)PD1 (median conc. 0.15 nM) and PDX (median conc. 0.072 nM) were quantified in 12–13 individuals (Figure 8). Despite high inter-individual variation, a trend toward higher levels (median concentration) of pro-inflammatory mediators, SPM precursors and SPMs from different enzymatic pathways (COX, 5-LOX, 15-LOX) and different PUFA (ARA, EPA, DHA) in plasma from septic shock patients compared to control was observed. This is consistent with earlier reports of an elevation of metabolites formed in the ARA cascade during inflammation [e.g., in a DSS-induced colitis model (Willenberg et al., 2015)] and could be caused by increased phospholipase A2 (PLA_2_) activity in response to the inflammatory stimuli e.g., in neutrophils (Levy et al., 2000). A slightly more pronounced elevation of SPM pathway markers, e.g., 17-HDHA (0.42 vs. 6.2 nM) compared to pro-inflammatory markers such as PGE_2_ (0.056 vs. 0.078 nM) could suggest an attempt of the body to resolve the inflammation; however, high mortality (72%) indicates failed resolution. In fact, despite the small sample size higher SPMs and their mono-hydroxylated precursors were found in non-survivors (*n* = 13) compared to survivors of septic shock (*n* = 5) (Figure 8). A similar observation was reported by Dalli et al., where higher plasma concentrations of SPMs including RvD1, RvD5, and (N)PD1 and pathway marker 17-HDHA in sepsis non-survivors were found. It should be noted that in this study several SPMs (e.g., PDX 0.004–0.008 nM) were found in a concentration below our LLOQ making it difficult to compare absolute concentrations (Dalli et al., 2017). Our study did not unveil an obvious correlation of SPM levels to clinical signs of inflammation, severity of sepsis or days of survival. However, possible correlations might be masked by the small and heterogeneous group of individuals: The patients were of different gender, age, health condition previous septic shock diagnosis and different pathogens were involved in the development of septic shock. No alteration of SPMs during inflammation or resolution phase was also observed in a human LPS induced sepsis model (Skarke et al., 2015) or SPMs were not detected in plasma samples from patients with hepatic failure despite showing clinical signs of inflammation (Toewe et al., 2018). In summary, although our study demonstrated the presence of detectable SPM concentrations in an exemplary cohort of extremely sick septic shock patients, it might not support a role of SPMs as biomarkers to predict the clinical outcome in sepsis.

## Conclusions

A new method for the detection of SPMs was developed by careful optimization of MS parameters in combination to an UHPLC chromatographic separation using one of the most sensitive—and commonly used for oxylipin quantification—instruments available. The resulting LOD were 0.18–2.7 pg on column corresponding to an LLOQ of 0.02–0.2 nM in biological samples such as plasma. SPMs were generally not detectable/quantifiable in plasma and serum of healthy individuals, while good recovery rates were found in spiked samples. These results strongly support findings that circulating levels of SPMs are very low, i.e., <0.1 nM in healthy subjects. In samples from patients with end stage renal disease (and peritonitis) or septic shock SPMs and precursors were detectable; however, not directly correlated with the health status and clinical outcome.

## Data Availability

All datasets generated for this study are included in the manuscript and/or the supplementary files.

## Author Contributions

LK and NHS designed study. LK, KR, AO, and NH conducted the current research under the supervision of NHS. J-MG, LB, and TD provided a standard compound. MB and SD provided clinical samples and designed clinical study. LK and NHS wrote the paper. NHS had primary responsibility for final content. All authors read and approved the final manuscript.

### Conflict of Interest Statement

The authors declare that the research was conducted in the absence of any commercial or financial relationships that could be construed as a potential conflict of interest. The reviewer NF declared a past co-authorship with several of the authors to the handling editor.

## Abbreviations

15(*R*)-LXA_4_: 5*S*,6*R*,15*R*-trihydroxy-7*E*,9*E*,11*Z*,13*E*-eicosatetraenoic acid
17(*R*)-RvD1: 7*S*,8*R*,17*R*-trihydroxy-4*Z*,9*E*,11*E*,13*Z*,15*E*,19*Z*-docosahexaenoic acid
18(*R*)-RvE3: 17*R*,18*R*- dihydroxy-5*Z*,8*Z*,11*Z*, 13*E*,15*E*-eicosapentaenoic acid
18(*S*)-RvE3: 17*R*, 18 *S*-dihydroxy-5*Z*,8*Z*,11*Z*,13*E*, 15*E*-eicosapentaenoic acid
6(*R*)-LXA_4_: 5*S*,6*R*,15*S*-trihydroxy-7*E*, 9*E*,11*Z*,13*E*-eicosatetraenoic acid
6(*S*)-LXA_4_: 5*S*,6*S*,15*S*-trihydroxy-7*E*,9*E*,11*Z*,13*E*-eicosatetraenoic acid
7(*S*)-MaR1: 7*S*,14 *S*-dihydroxy-4*Z*,8*E*, 10*E*,12*Z*,16*Z*,19*Z*-docosahexaenoic acid
ARA: arachidonic acid (20:4 n6)
CAD: collision activated dissociation
CID: collision induced dissociation
CE: collision energy
CXP: collision cell exit potential
DHA: docosahexaenoic acid (22:6 n3)
DP: declustering potential
EP: entrance potential
EPA: eicosapentaenoic acid (20:5 n3)
ESI: electrospray ionization
FIA: flow injection analysis
HDHA: hydroxy docosahexaenoic acid
HEPE: hydroxy eicosapentaenoic acid
HETE: hydroxy eicosatetraenoic acid
HPLC: high performance liquid chromatography
IS: internal standard
LC: liquid chromatography
LLOQ: lower limit of quantification
LOD: limit of detection
LX: lipoxin(s)
LXA_5_: 5*S*,6*R*,15*S*-trihydroxy-7*E*,9*E*,11*Z*,13*E*,17*Z*-eicosapentaenoic acid
LXB_4_: 5*S*,14*R*,15*S*-trihydroxy-6*E*, 8*Z*,10*E*, 12*E*-eicosatetraenoic acid
MaR: maresin(s)
MaR1: 7*R*, 14*S*-dihydroxy-4*Z*,8 *E*,10*E*,12*Z*,16*Z*,19*Z*-docosahexaenoic acid
MS: mass spectrometry/ mass spectrometric
n3/n6: omega-3/omega-6
(N)PD1: 10*R*, 17*S*-dihydroxy-4*Z*,7*Z*, 11*E*,13*E*,15*Z*,19*Z*-docosahexaenoic acid
PD: peritoneal dialysis
PDX: 10*S*,17*S*-dihydroxy-4*Z*,7*Z*,11*E*,13*Z*,15*E*,19*Z*-docosahexaenoic acid
PUFA: polyunsaturated fatty acid
RP: reversed phase
Rv: resolvin(s)
RvD1: 7*S*,8*R*,17*S*-trihydroxy-4*Z*,9*E*,11*E*,13*Z*,15*E*,19*Z*-docosahexaenoic acid
RvD2: 7*S*, 16*R*,17*S*-trihydroxy-4*Z*, 8*E*,10*Z*, 12*E*,14*E*, 19*Z*-docosahexaenoic acid
RvD3: 4*S*,11*R*,17*S*-trihydroxy-5*Z*,7*E*,9*E*,13*Z*,15*E*,19*Z*-docosahexaenoic acid
RvD5: 7*S*, 17*S*-dihydroxy-4*Z*,8*E*,10*Z*, 13*Z*,15*E*,19*Z*-docosahexaenoic acid
RvE1: 5*S*,12*R*, 18*R*-trihydroxy-6*Z*,8*E*,10*E*,14*Z*,16*E*-eicosapentaenoic acid
RvE2: 5*S*,18*R*-dihydroxy-6*E*,8*Z*,11*Z*,14*Z*,16*E*-eicosapentaenoic acid
S/N: signal-to-noise-ratio
SPM: specialized pro-resolving mediator
SRM: selected reaction monitoring
ULOQ: upper limit of quantification.
