# Supplementary material for: Development of an Optimized LC-MS Method for the Detection of Specialized Pro-Resolving Mediators in Biological Samples
Source: Front Pharmacol. 2019 Mar 7;10:169. doi: 10.3389/fphar.2019.00169 (PMC6416208; doi:10.3389/fphar.2019.00169)
Supplement: Supplementary file 1 [file Presentation_1.pdf]

Supplementary information

Development of an optimized LC-MS method for the detection of specialized pro-resolving mediators in biological samples

L. Kutzner, K. M. Rund, A. I. Ostermann, N. M. Hartung, J.-M. Galano, L. Balas, T. Durand, M. S. Balzer, S. David and N. H. Schebb

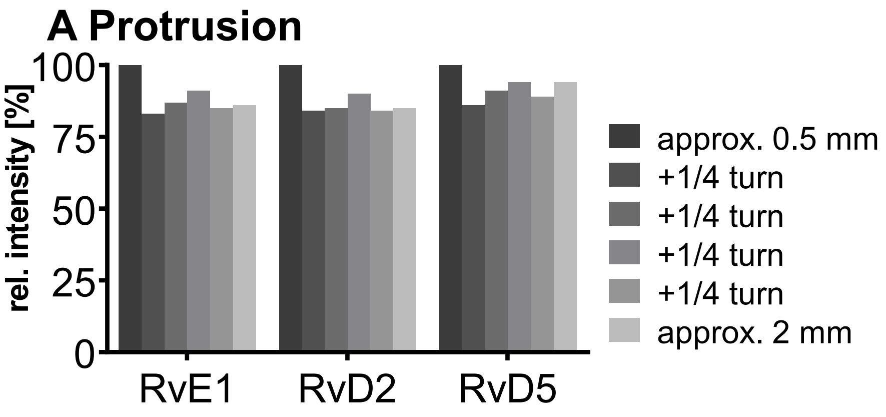

Fig. S1: Optimization of source parameters in SRM mode for compounds RvE1, RvD2 and RvD5. (**A**) Electrode protrusion was adjusted between approx. 0.5 mm and 2 mm, (**B**) probe position along the y-axis was optimized between 0 mm (farthest position relative to orifice) and 5 mm, (**C**) probe position along the x-axis was optimized between 2.5 mm and 7.5 mm (5 mm = center position relative to orifice), (**D**) nebulizer gas (GS1) was optimized between 30 psi and 70 psi, (**E**) auxiliary (heater) gas (GS2) was optimized between 40 psi and 70 psi at constant source temperature of 475 °C. Optimization was carried out in FIA mode (100 nM).

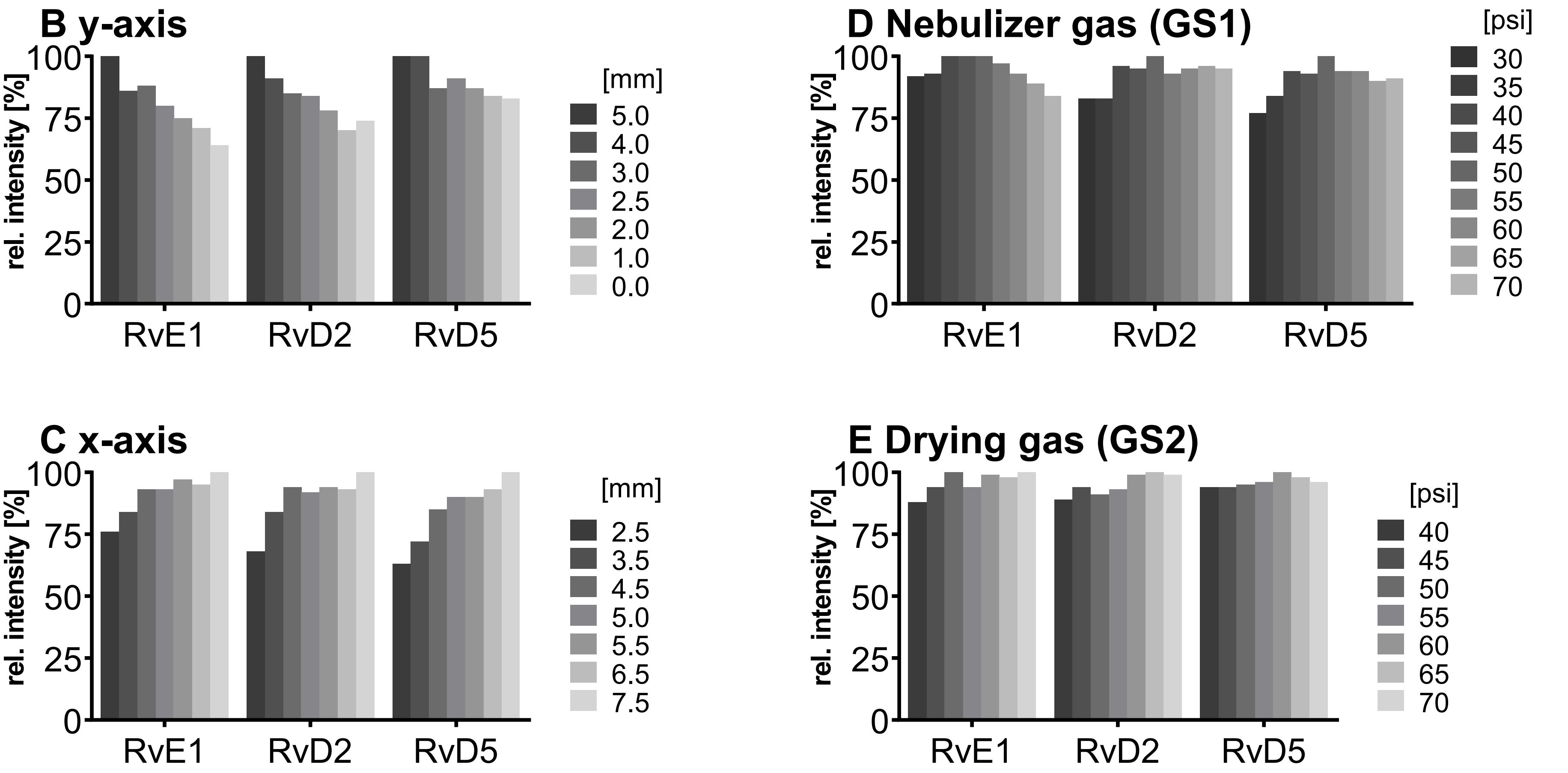

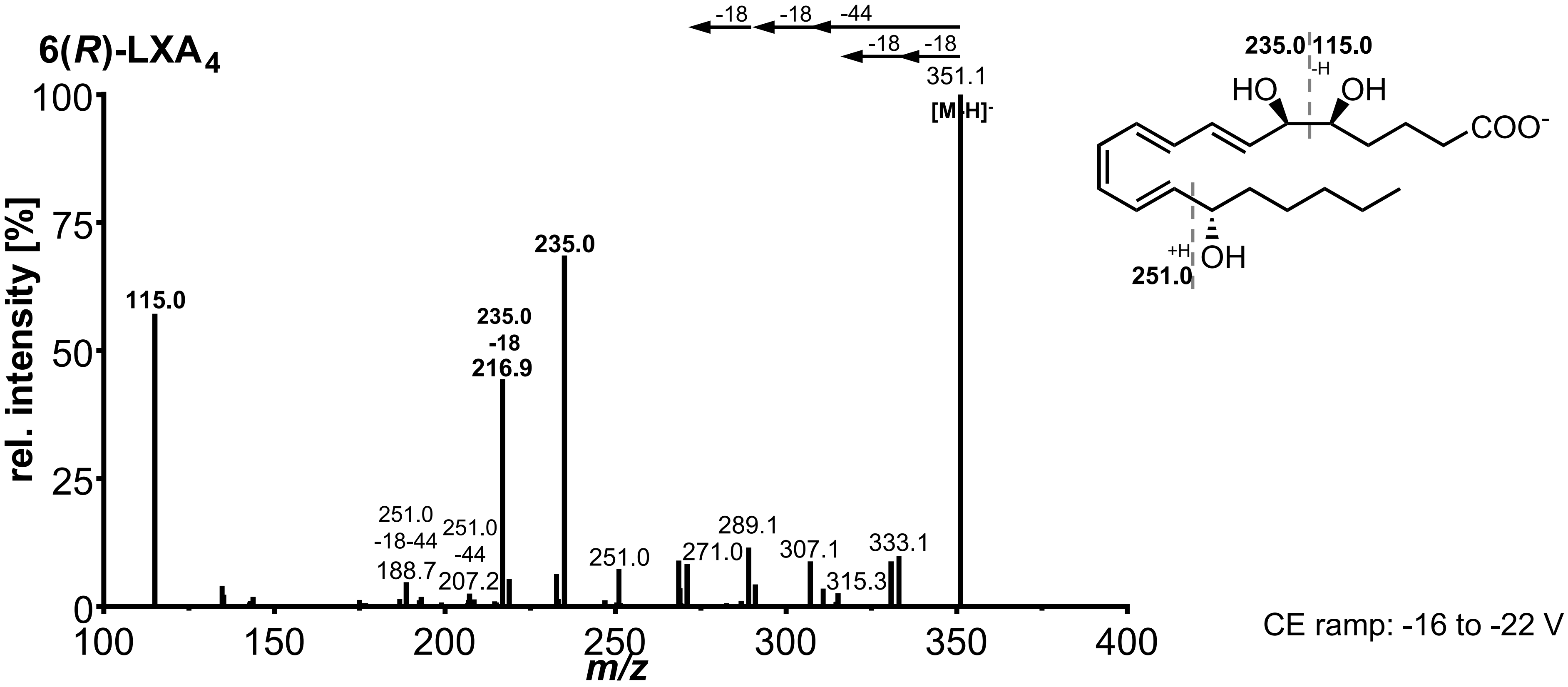

Fig. S2: Product ion spectra of ARA, EPA and DHA derived SPMs. The fragment spectra were aquired via CID applying a collision energy (CE) ramp.

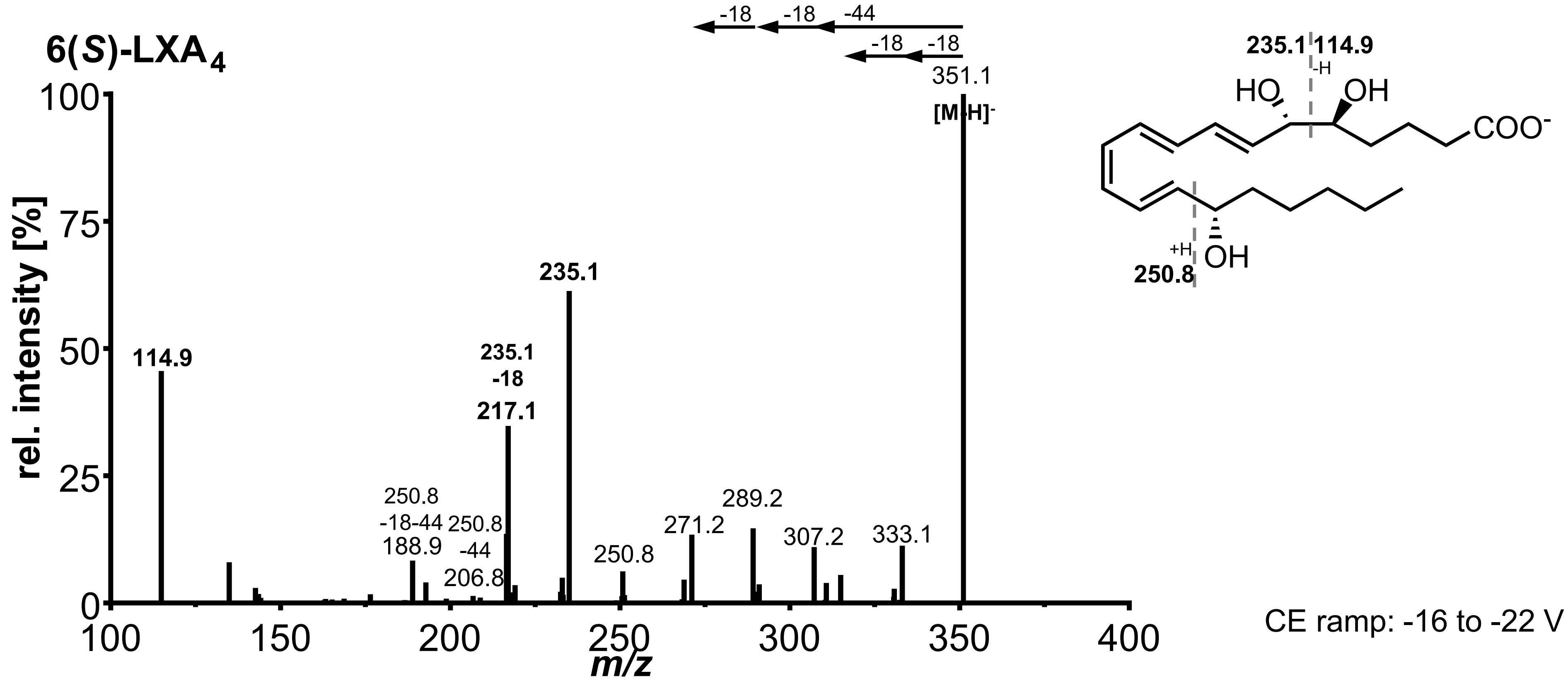

Fig. S2: *Continued*.

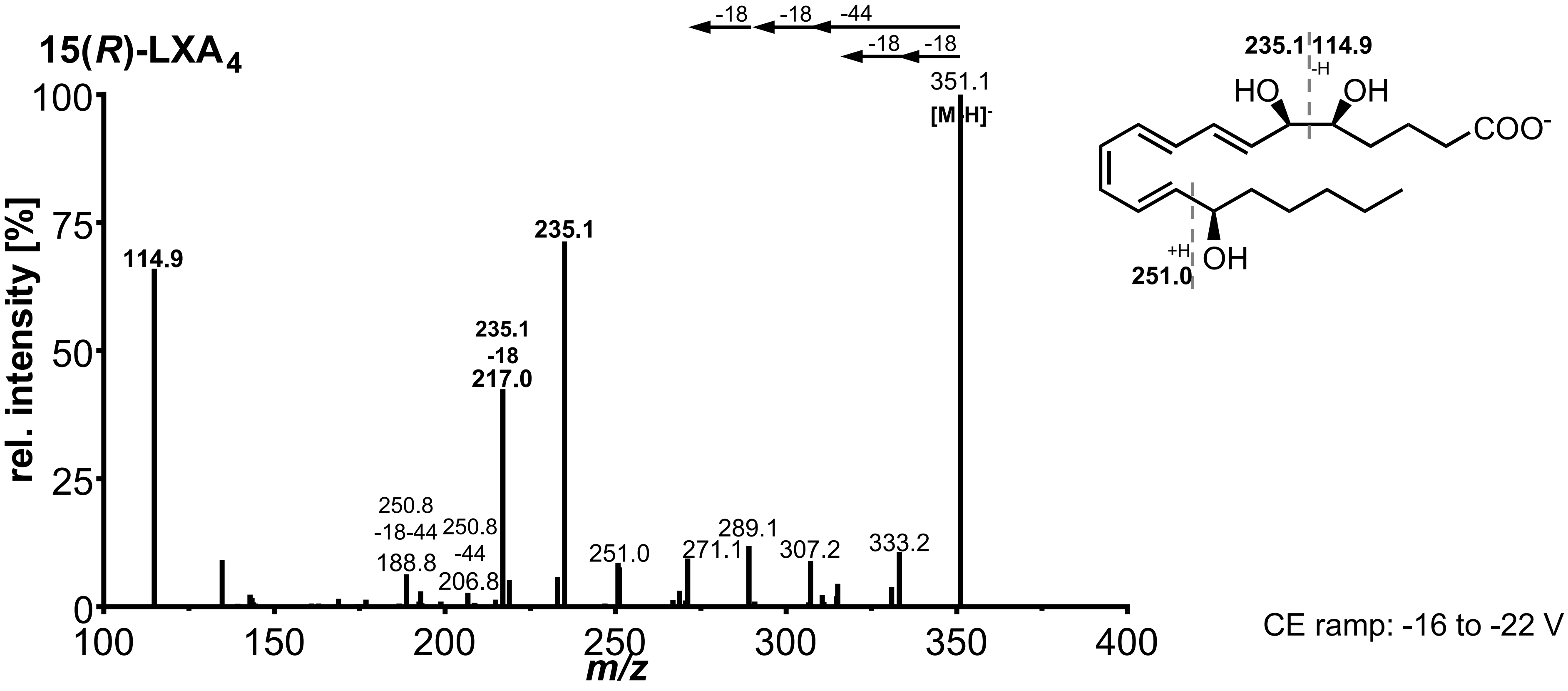

Fig. S2: *Continued*.

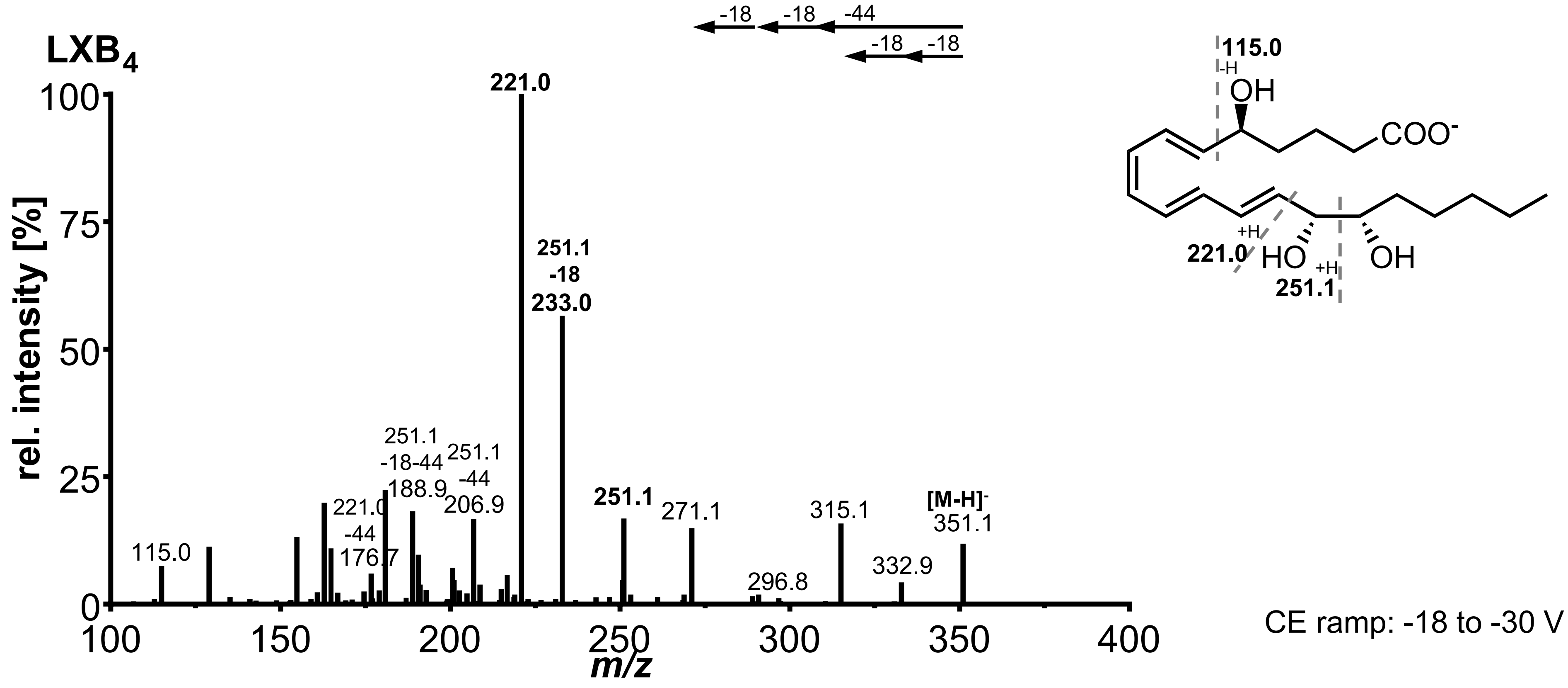

Fig. S2: *Continued*.

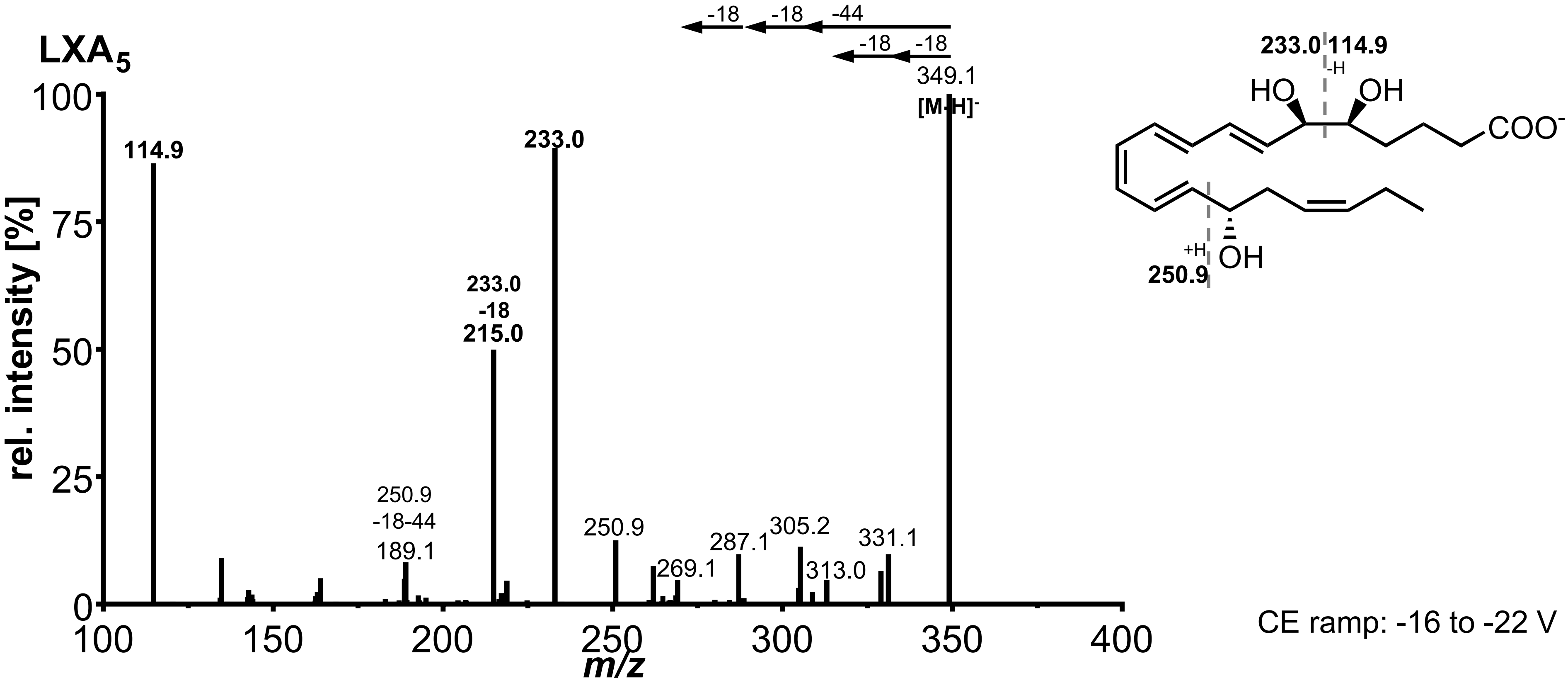

Fig. S2: *Continued*.

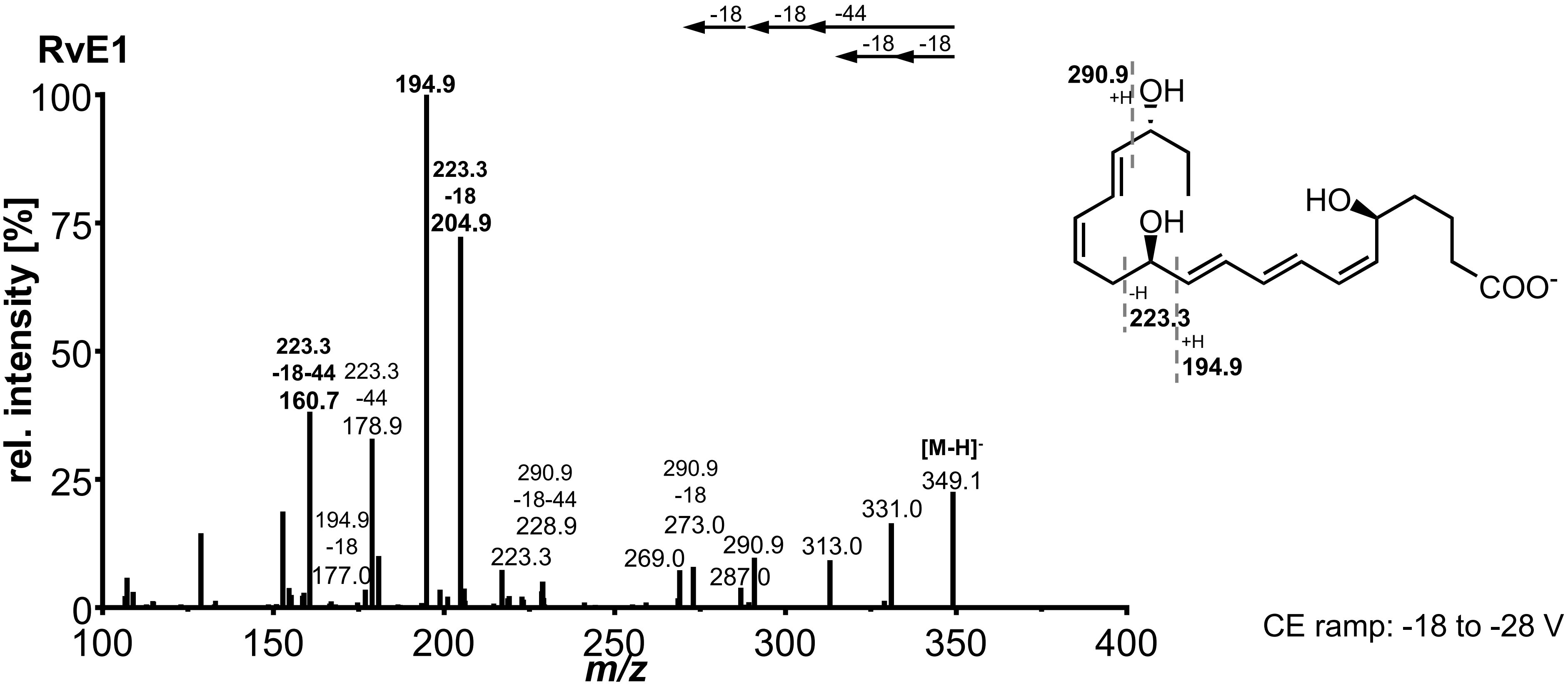

Fig. S2: *Continued*.

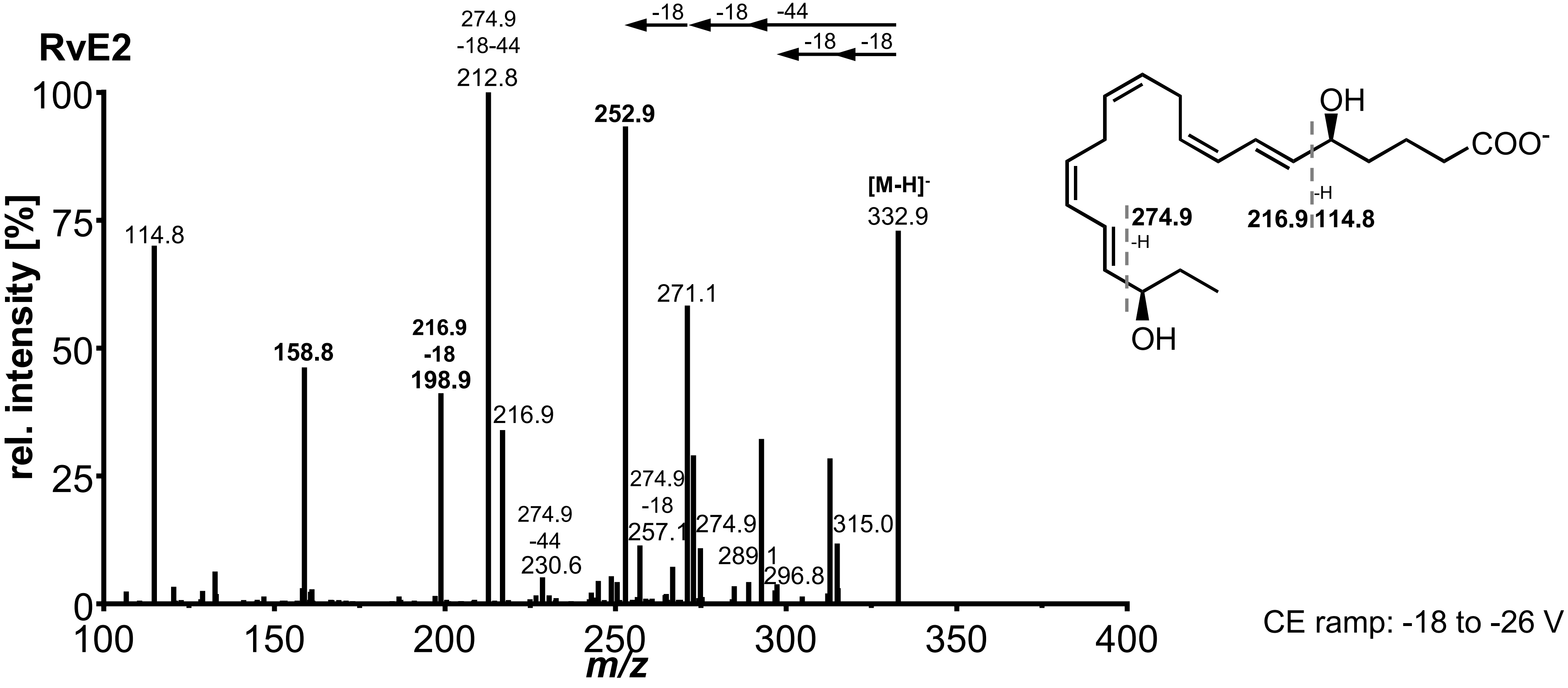

Fig. S2: *Continued*.

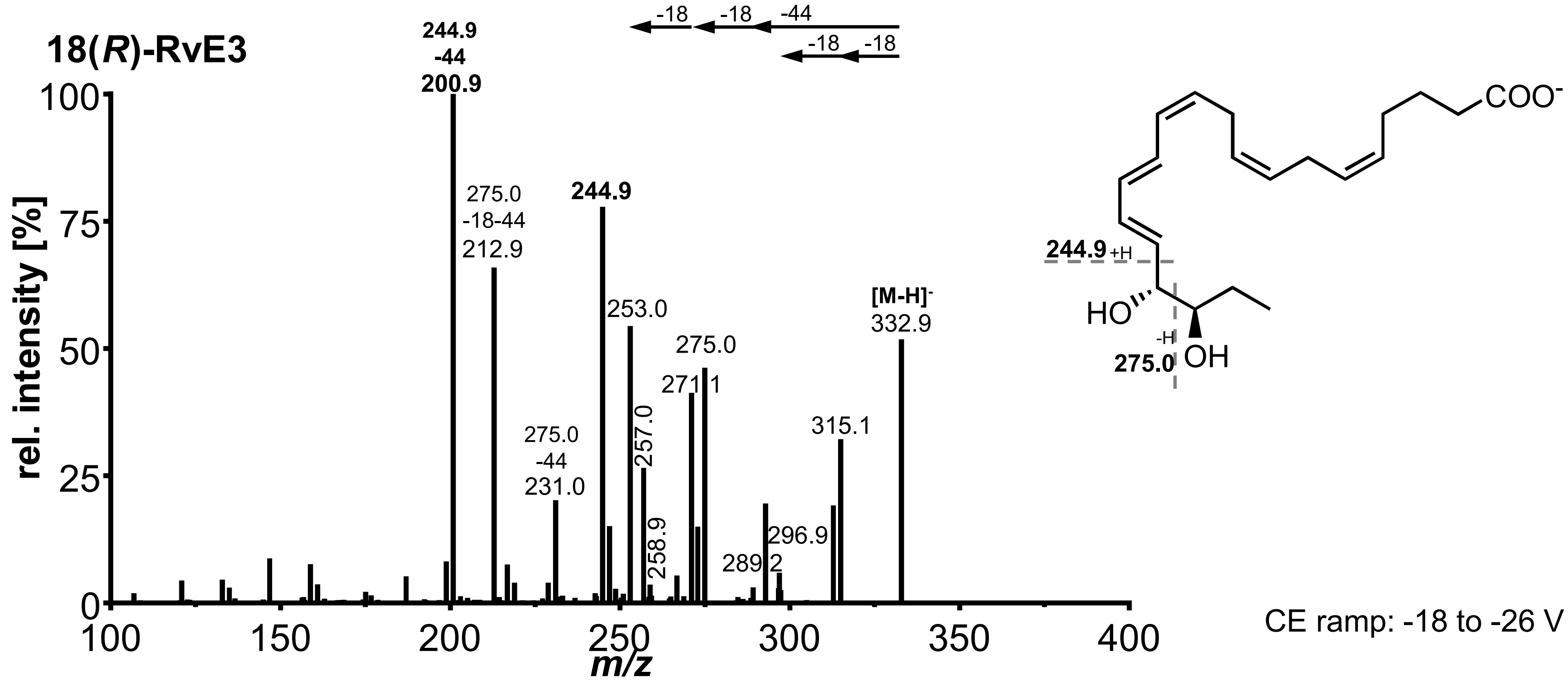

Fig. S2: *Continued*.

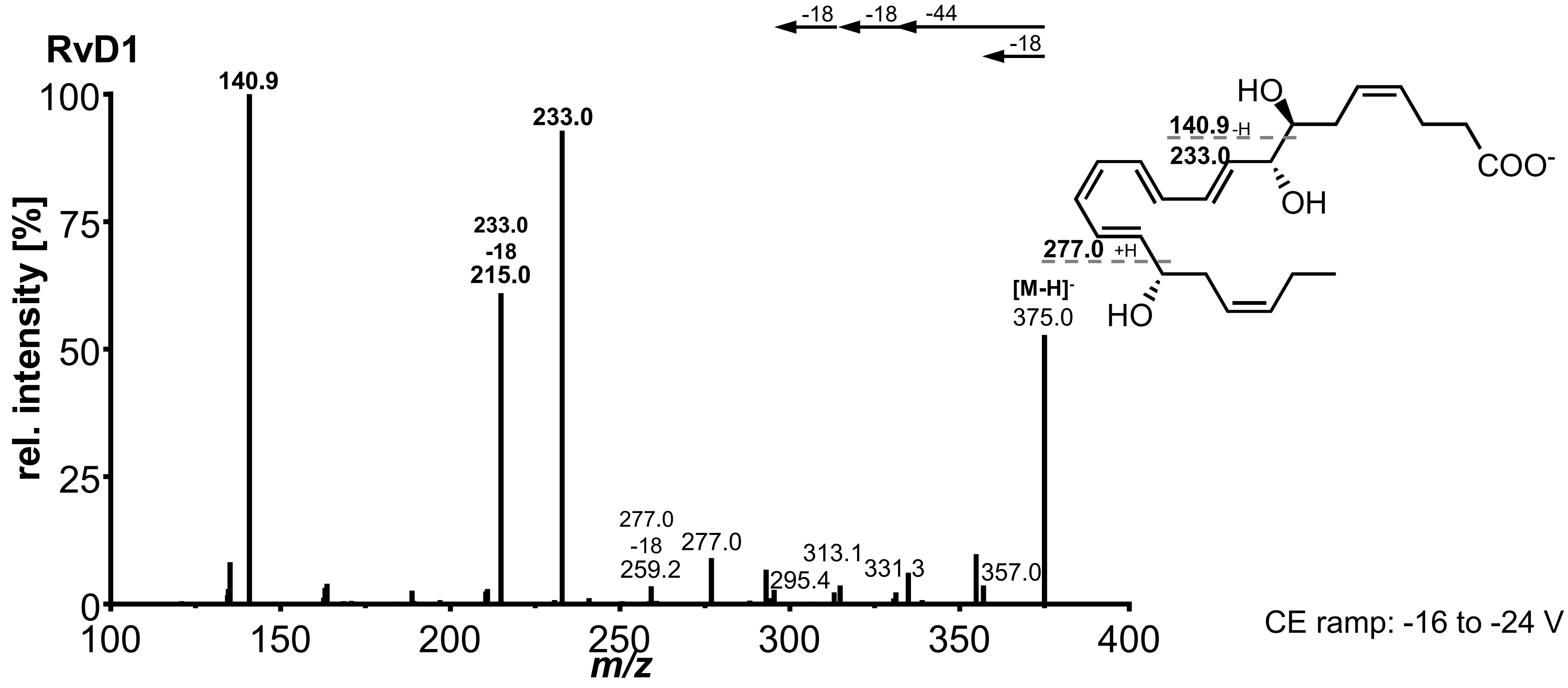

Fig. S2: *Continued*.

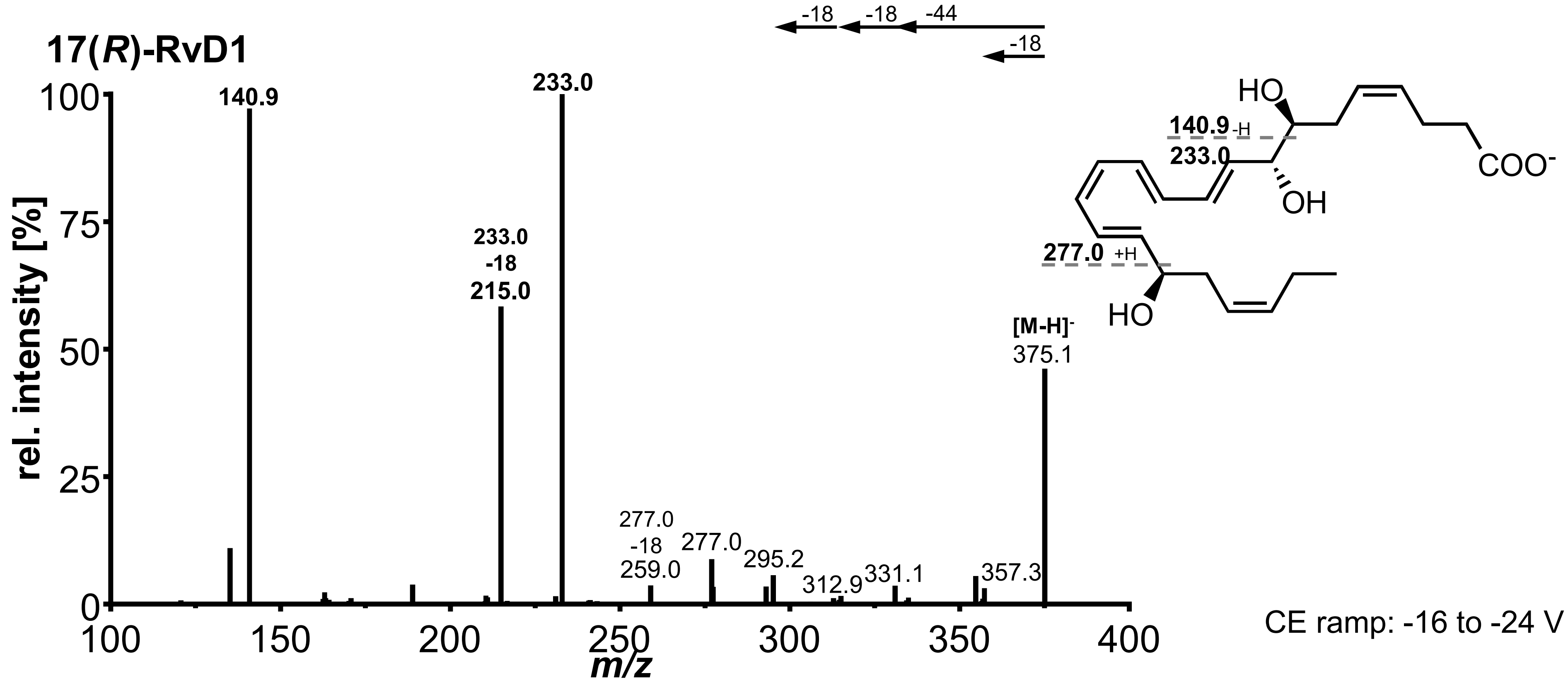

Fig. S2: *Continued*.

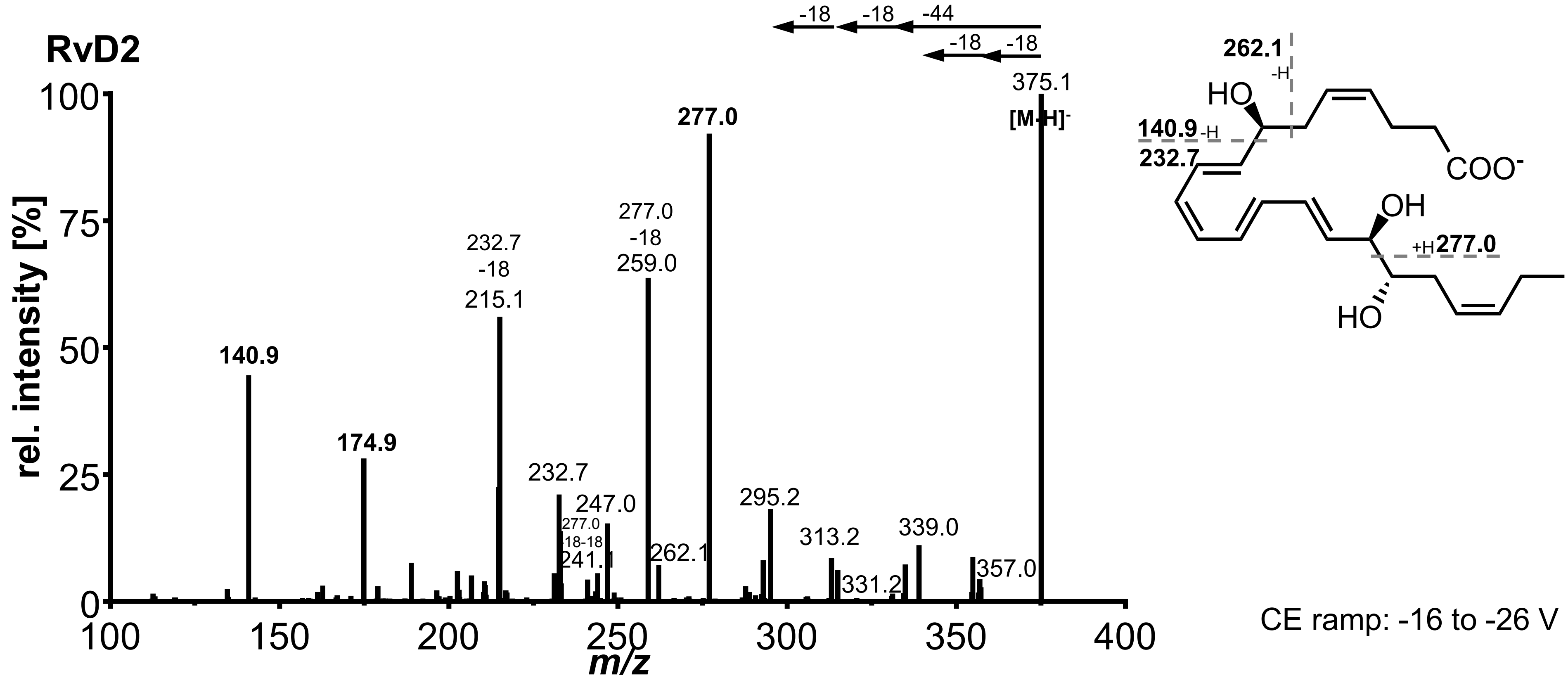

Fig. S2: *Continued*.

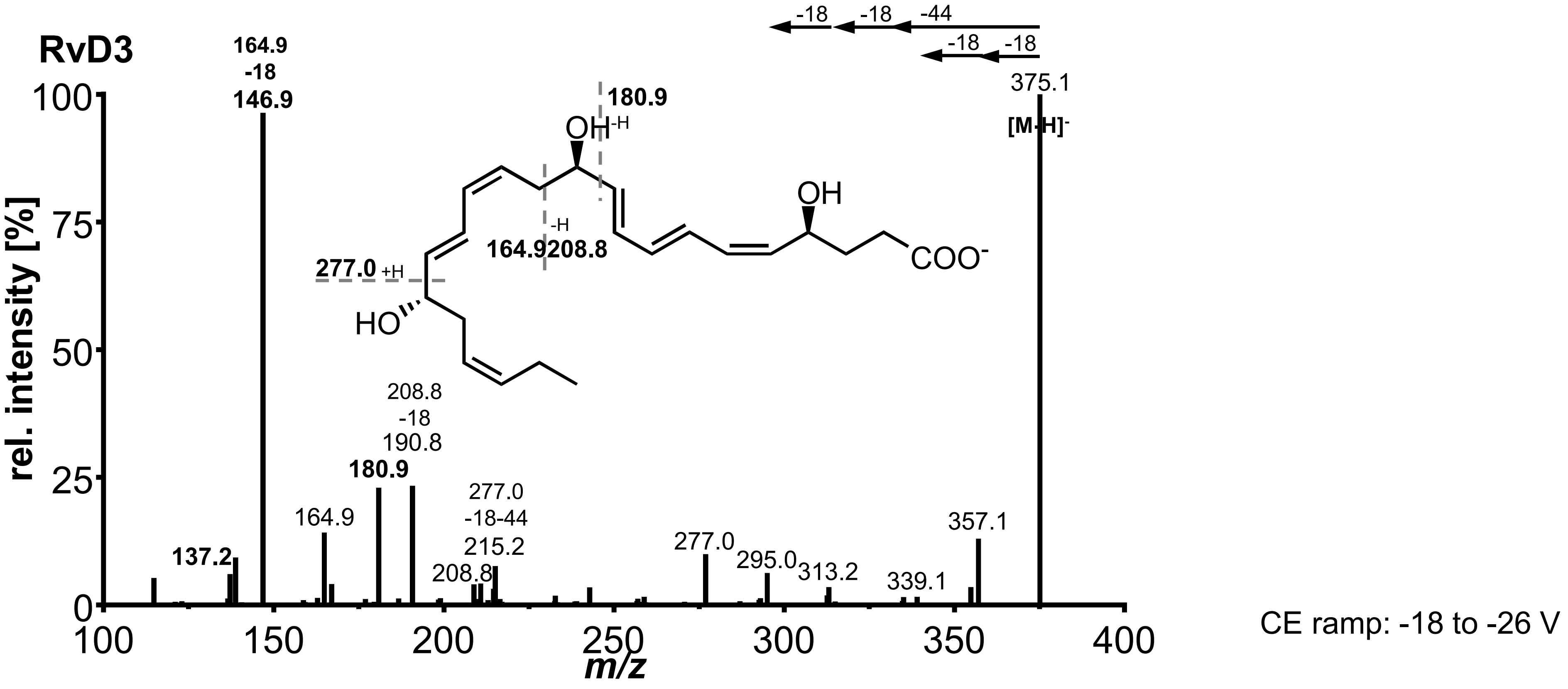

Fig. S2: *Continued*.

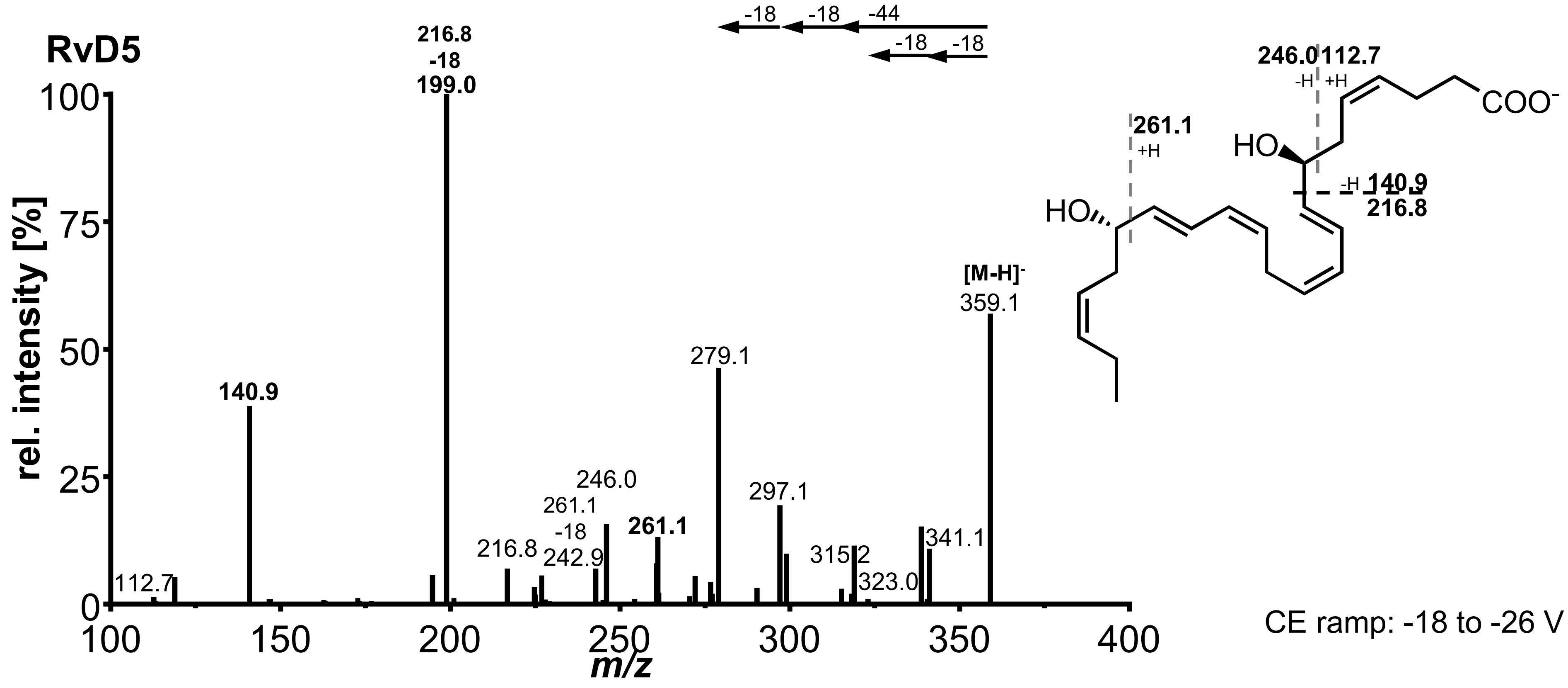

Fig. S2: *Continued*.

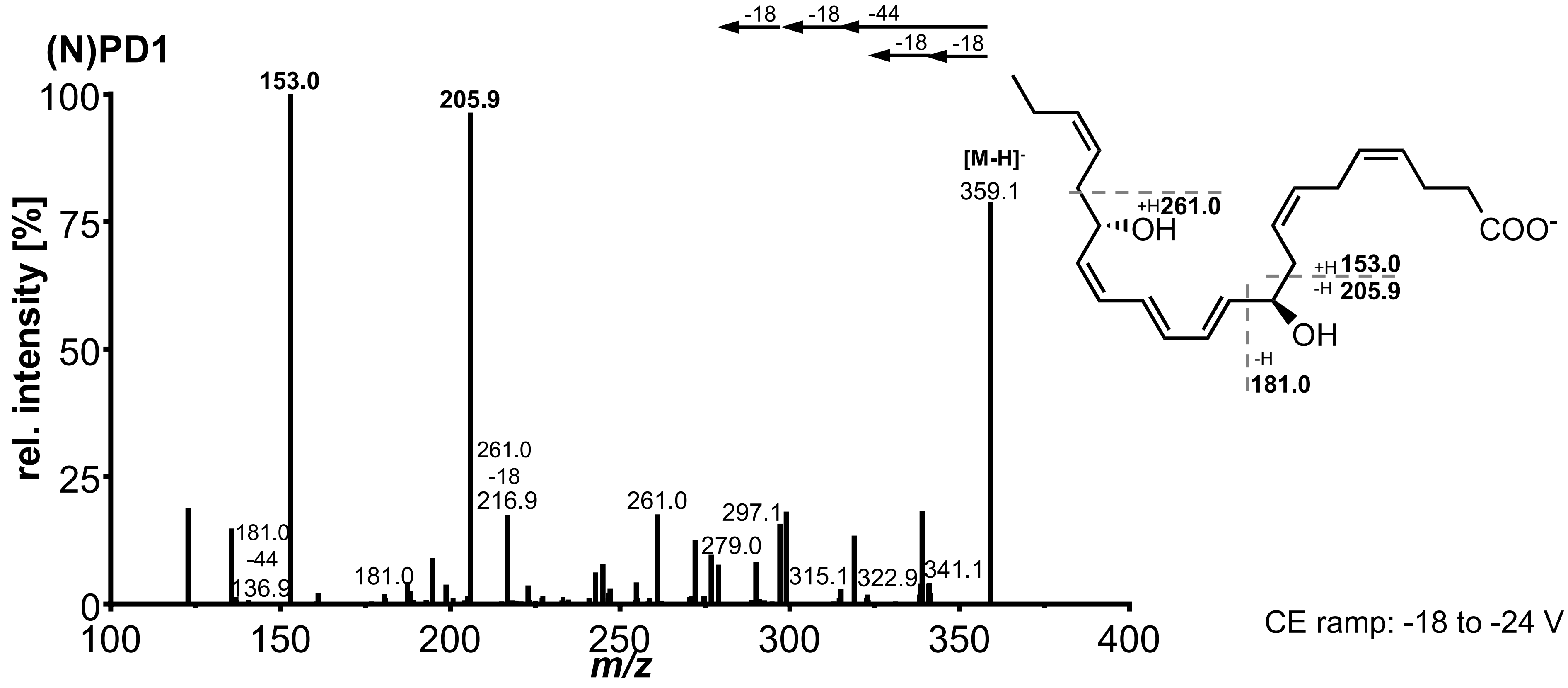

Fig. S2: *Continued*.

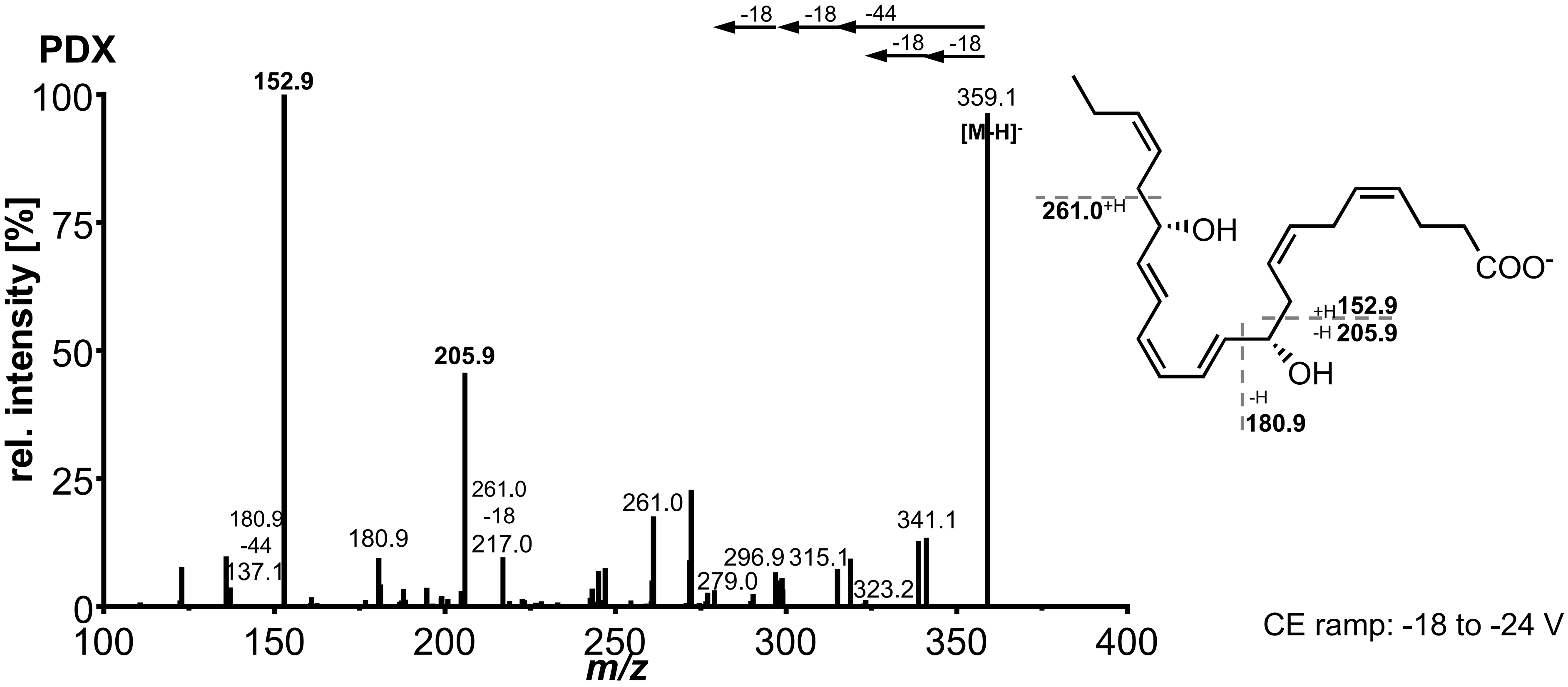

Fig. S2: *Continued*.

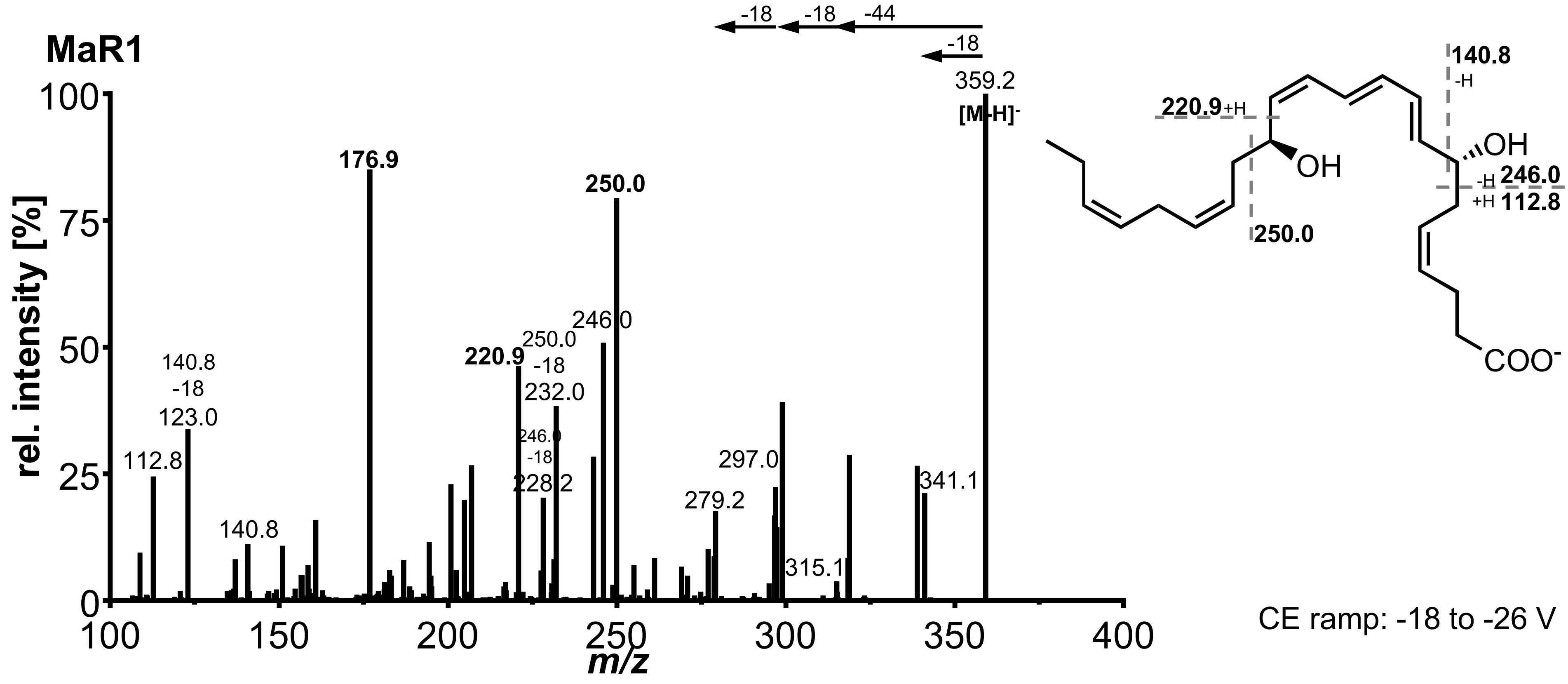

Fig. S2: *Continued*.

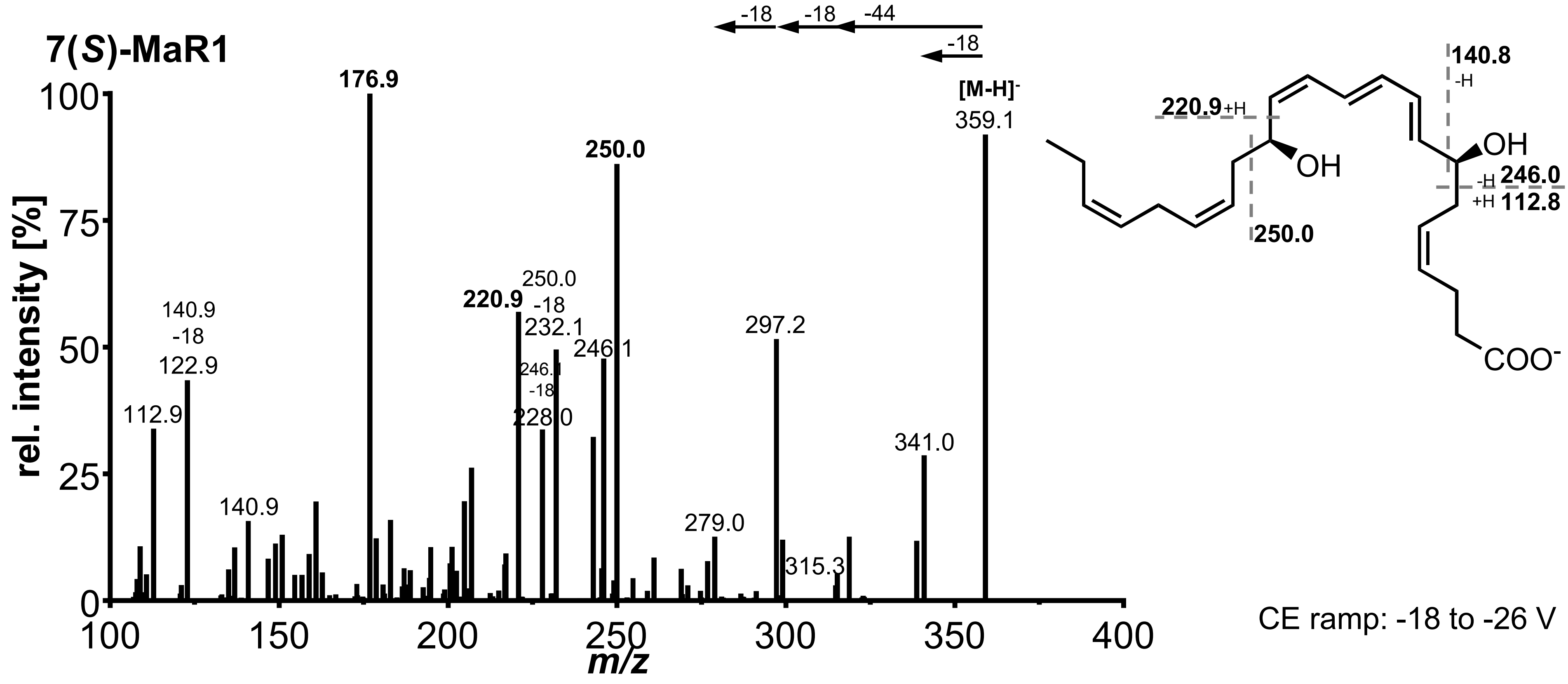

Fig. S2: *Continued*.

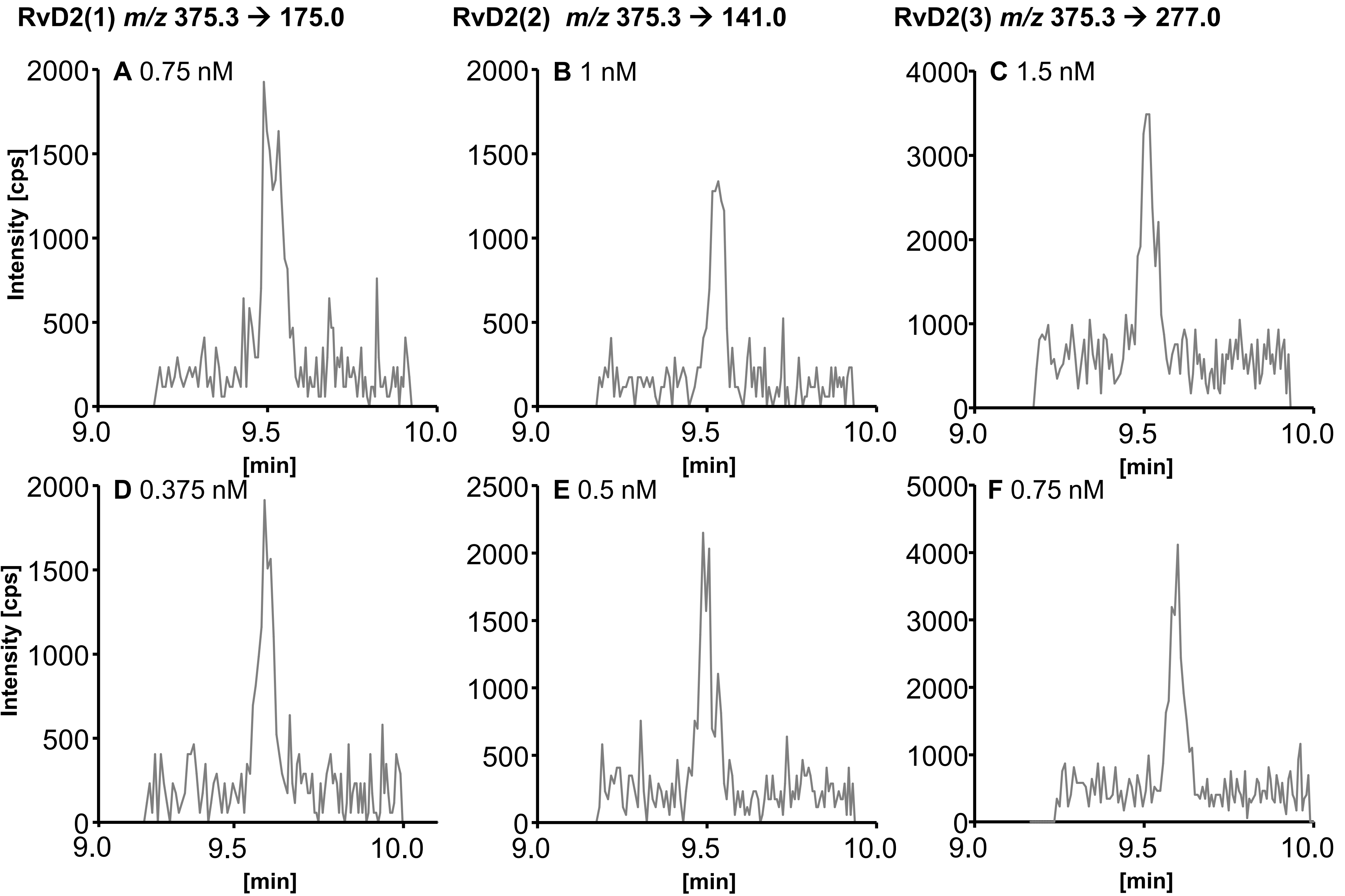

Fig. S3: Lower limits of quantification (LLOQ) for resolvin D2 on all three transitions (*m/z* 141.0, *m/z* 175.0, *m/z* 277.0). With an injection volume of 10 µL (**D-F**) an improvement of LLOQs can be achieved compared to injection volume of 5 µL (**A-C**).

(A) RvE1 *m/z* 349.3 → 195.0

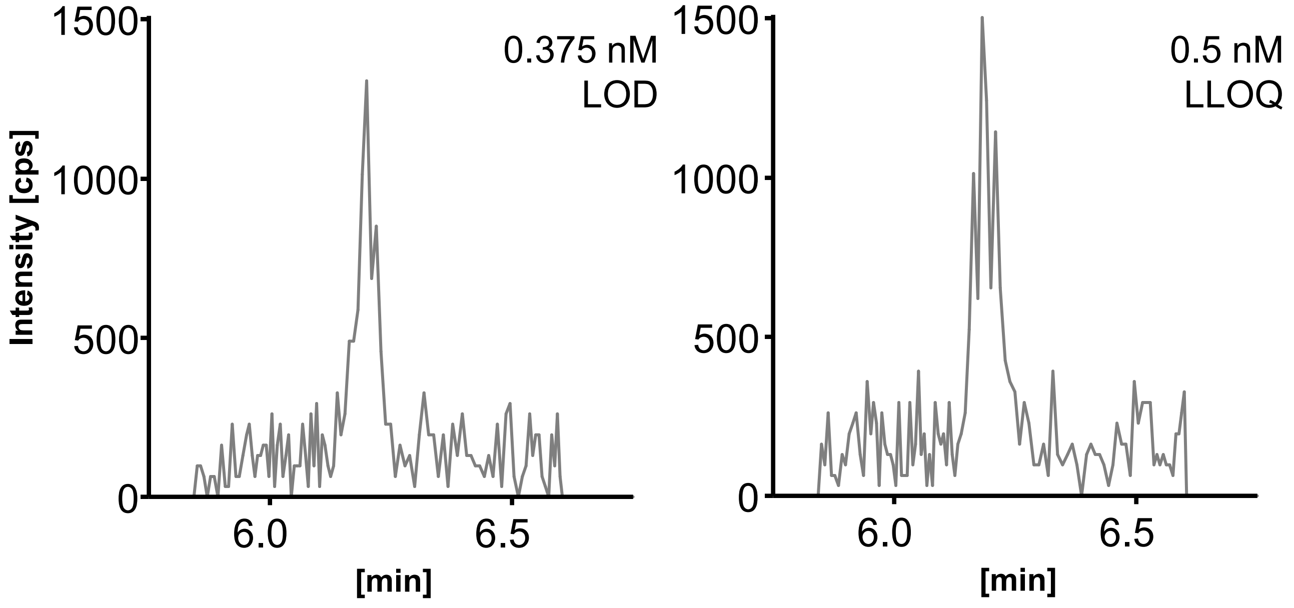

(B) RvD2 *m/z* 375.3 → 175.0

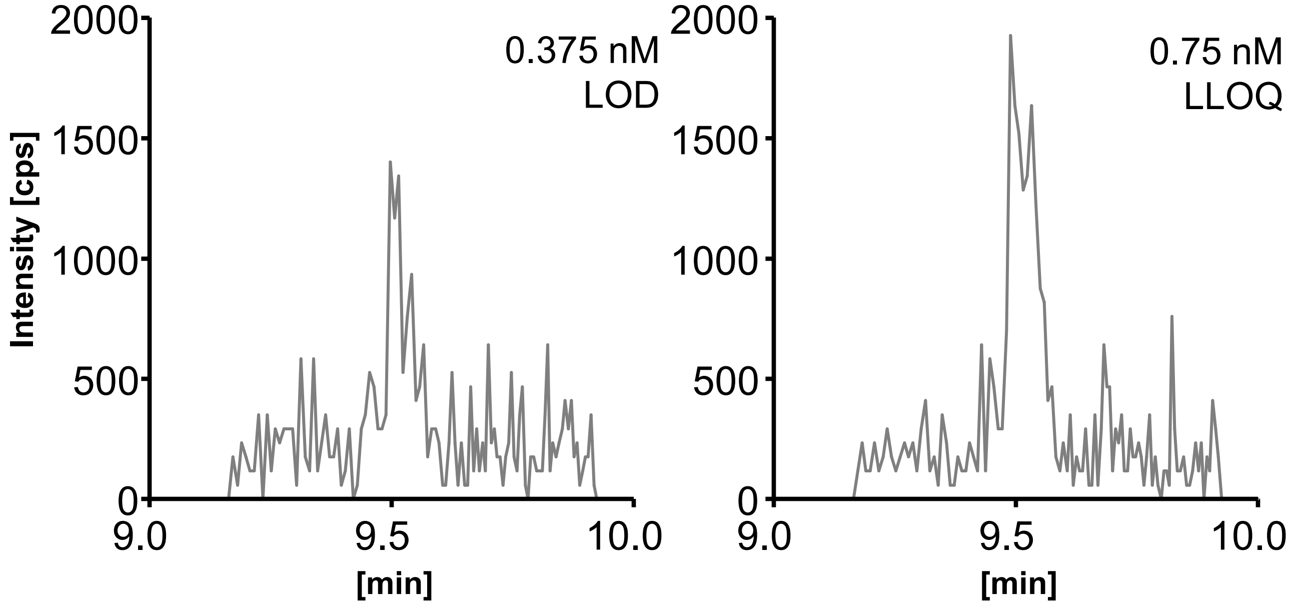

Fig. S4: Chromatographic peaks for the concentrations representing the limits of detection (LOD) and lower limits of quantification (LLOQ) for structurally representative SPMs for 5 µL injection volume. (**A**) EPA-derived trihydroxy-FA RvE1, (**B**) DHA-derived trihydroxy-FA RvD2, (**C**) ARA-derived trihydroxy-FA 6(*R*)-LXA_4, (**D**) DHA-derived dihydroxy-FA RvD5, (**E**) DHA-derived dihydroxy-FA 7(*S*)-MaR1 and (**F**) DHA-derived protectin PDX.

(C) 6(*R*)-LXA_4 *m/z* 351.2 → 115.2

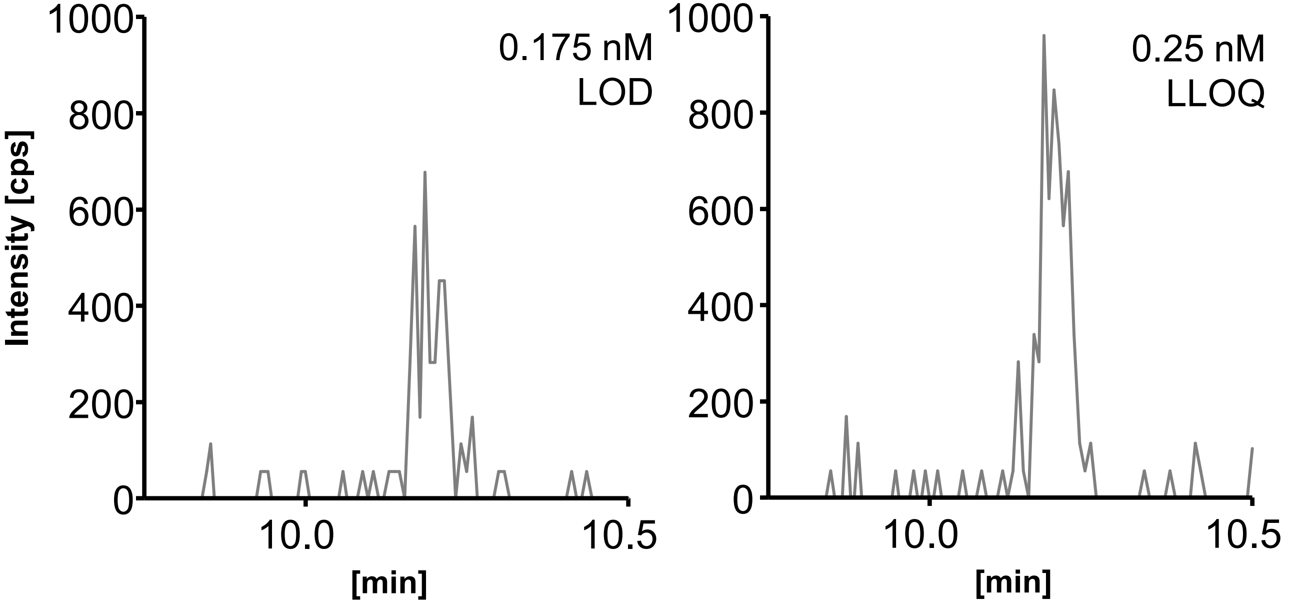

Fig. S4: *Continued*.

(D) RvD5 *m/z* 359.1 → 199.1

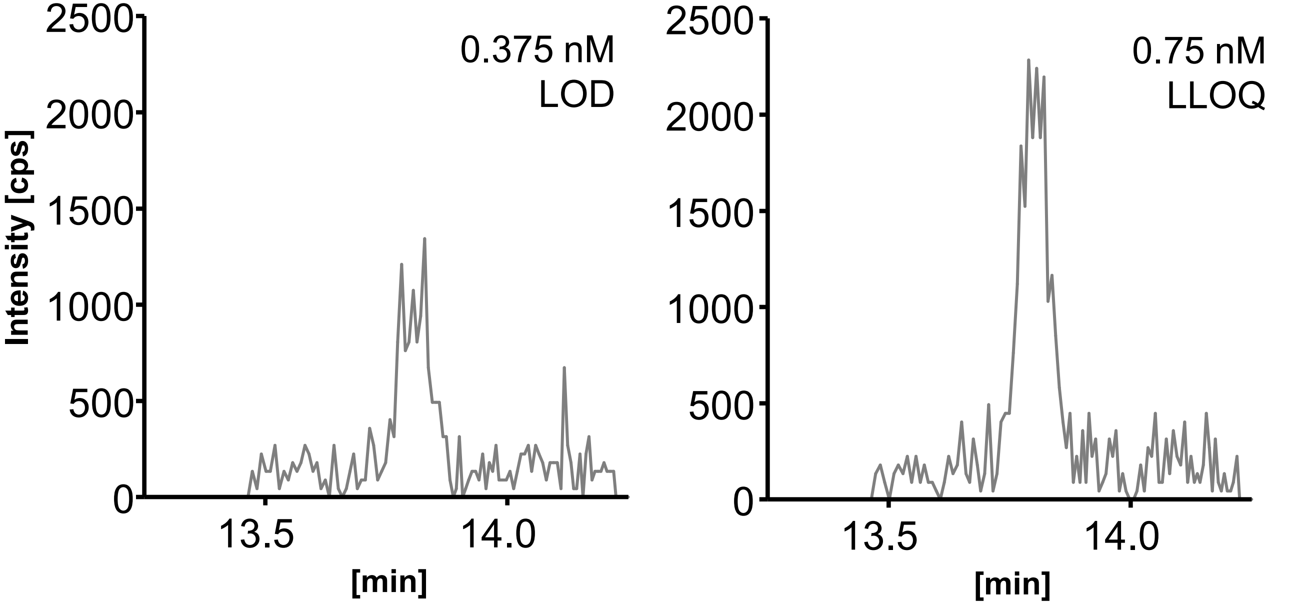

(E) 7(*S*)-MaR1 *m/z* 359.1 → 250.2

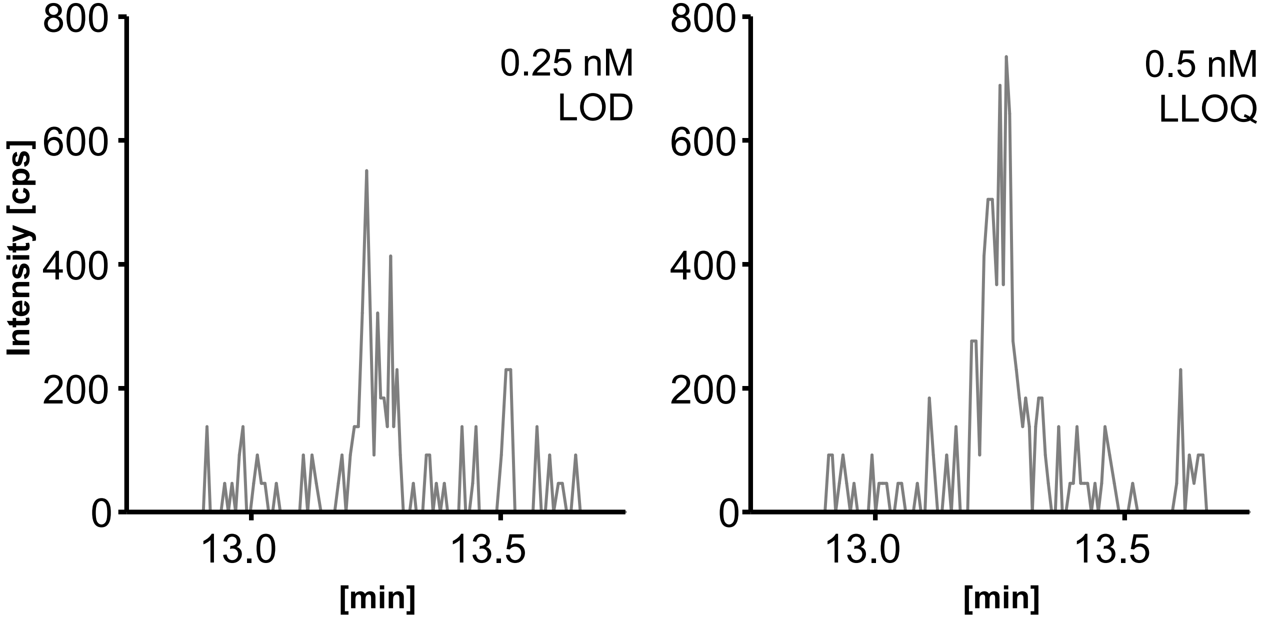

Fig. S4: *Continued*.

(F) PDX *m/z* 359.1 → 153.1

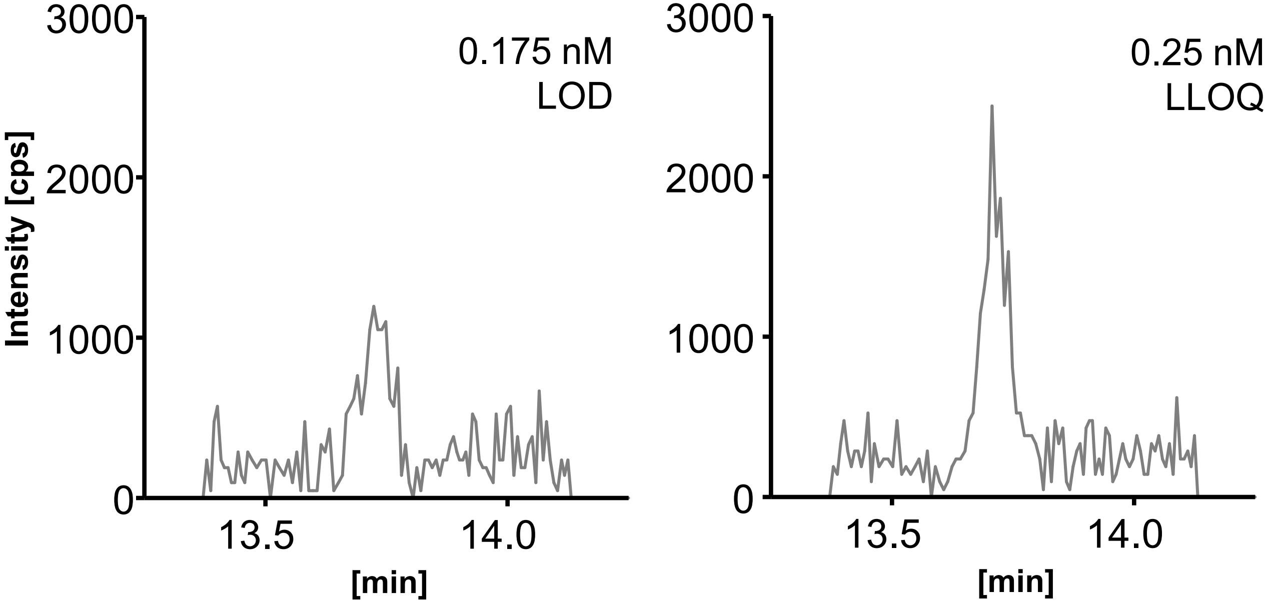

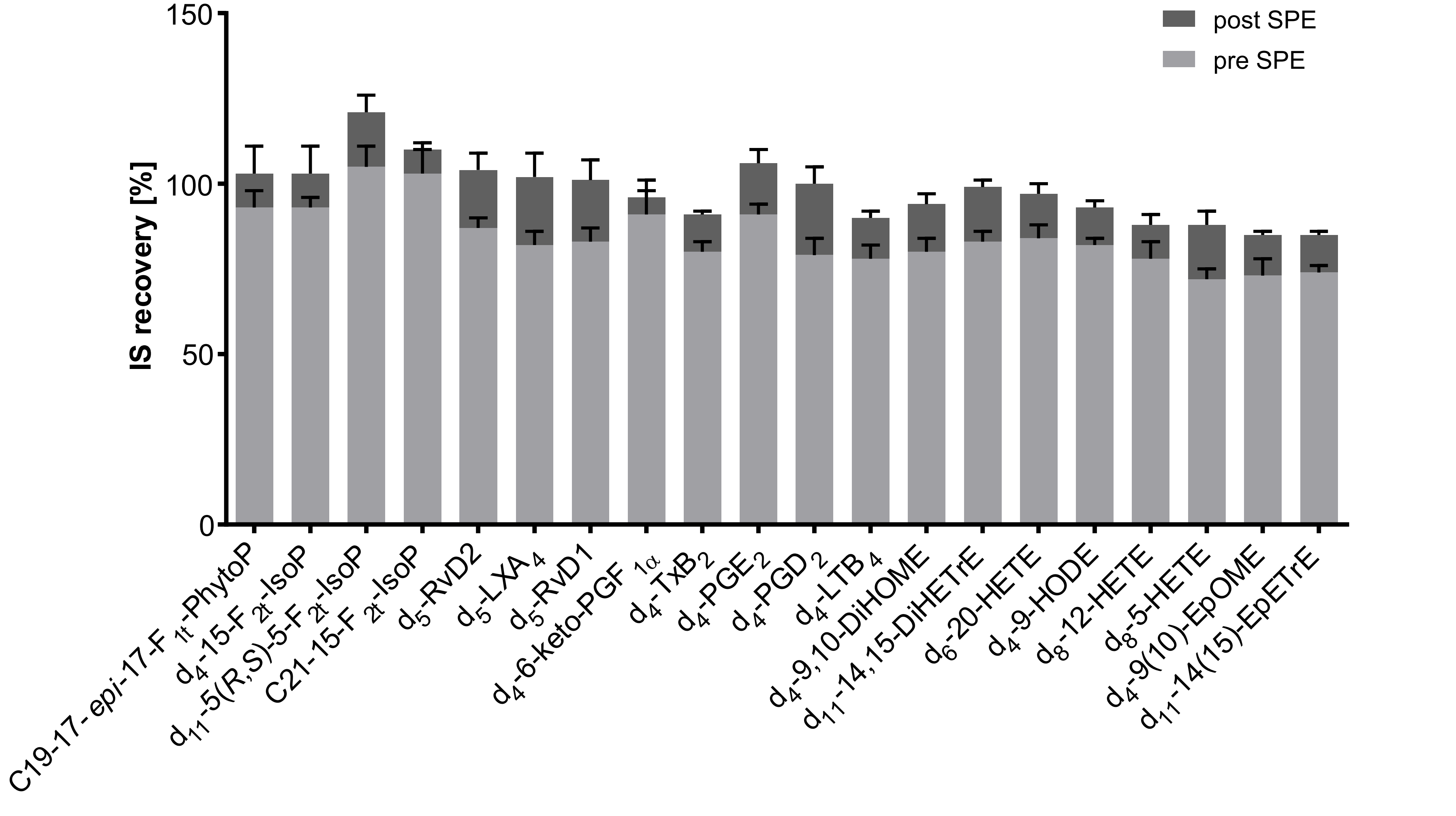

Fig. S5: Recovery of all 20 deuterated internal standards (IS) used for quantification of oxylipins in 500 µL serum. Recovery of IS 1 (added directly at the beginning of sample preparation) was determined utilizing 1-(1-(ethylsulfonyl)piperidin-4-yl)-3-(4-(trifluoromethoxy)phenyl)urea as IS 2 (added after sample preparation directly before measurement). Evaluation of ion suppression by direct comparison of IS addition at the beginning of sample preparation and directly before reconstitution of sample extract after SPE.

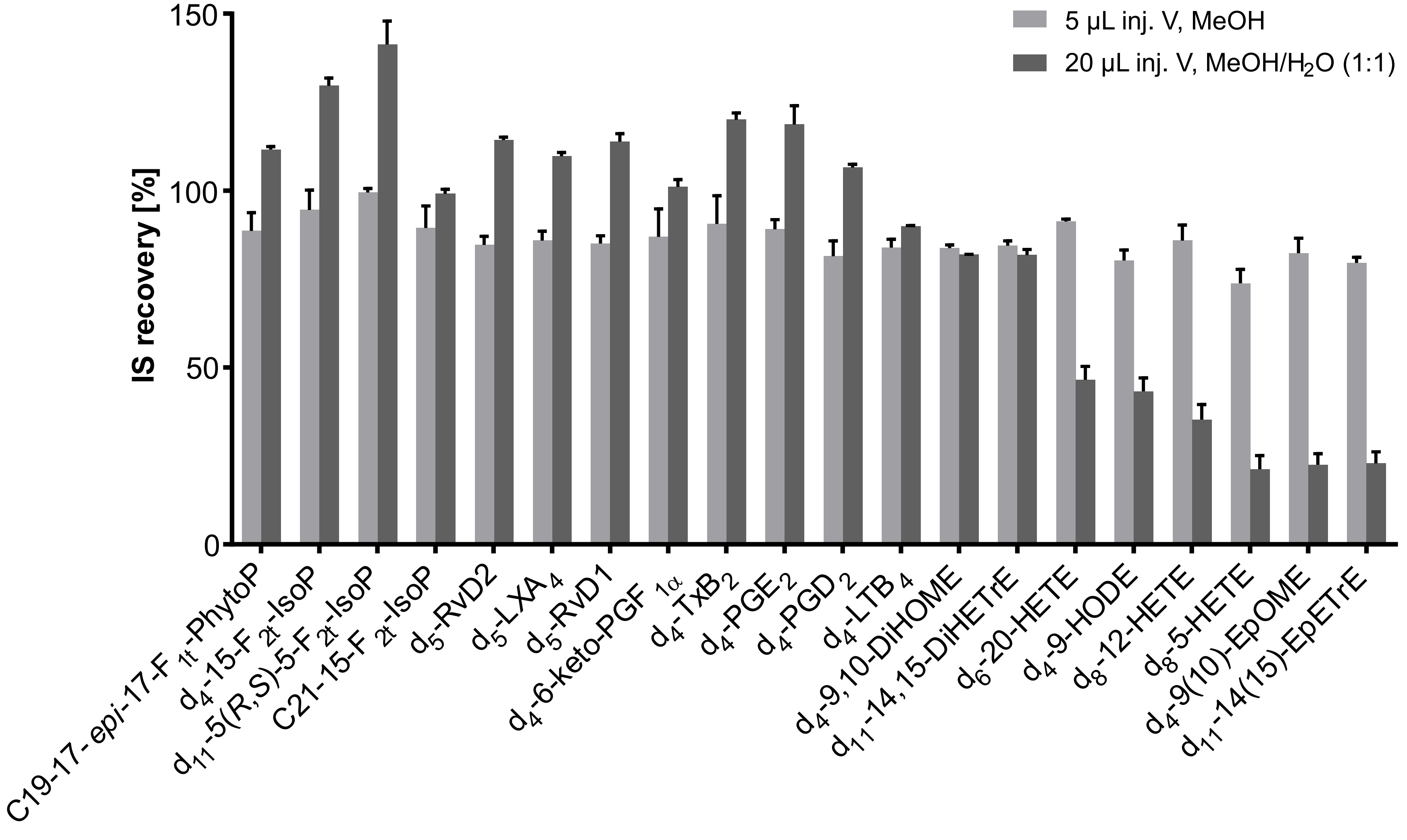

Fig. S6: Recovery of all 20 deuterated internal standards (IS) used for quantification of oxylipins in plasma. Insufficient recovery rates for less polar IS when sample extract was reconstituted in 50:50 MeOH:H_2O and 20 µL were injected; good recovery rates when sample extract was reconstituted in 100% MeOH and 5 µL were injected. Recovery of IS 1 (added directly at the beginning of sample preparation) was determined utilizing 1-(1-(ethylsulfonyl)piperidin-4-yl)-3-(4-(trifluoromethoxy)phenyl)urea as IS 2 (added after sample preparation directly before measurement).

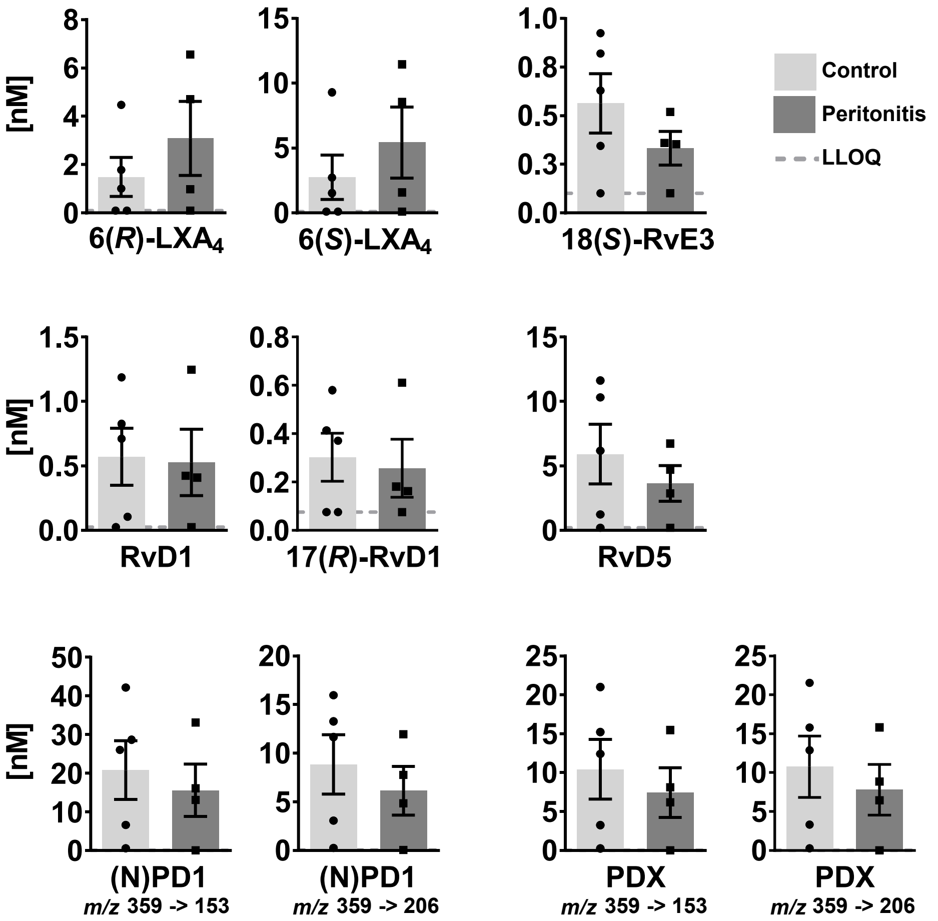

Fig. S7: Concentration of SPMs in serum from patients with end stage renal disease treated with peritoneal dialysis (PD) with (peritonitis, n=4-5) or without (control, n=4-5) acute inflammation. Shown are concentrations in nM as individual values and mean ± SEM of SPMs derived from ARA, EPA and DHA that were not displayed in figure 7. For concentrations <LLOQ, the LLOQ is given. The LLOQ is indicated as dotted line. For (N)PD1 and PDX two transitions are displayed. PDX shows good agreement between determined concentrations for both transitions, while for (N)PD1 the transitions lead to different apparent concentrations, probably due to matrix interferences.

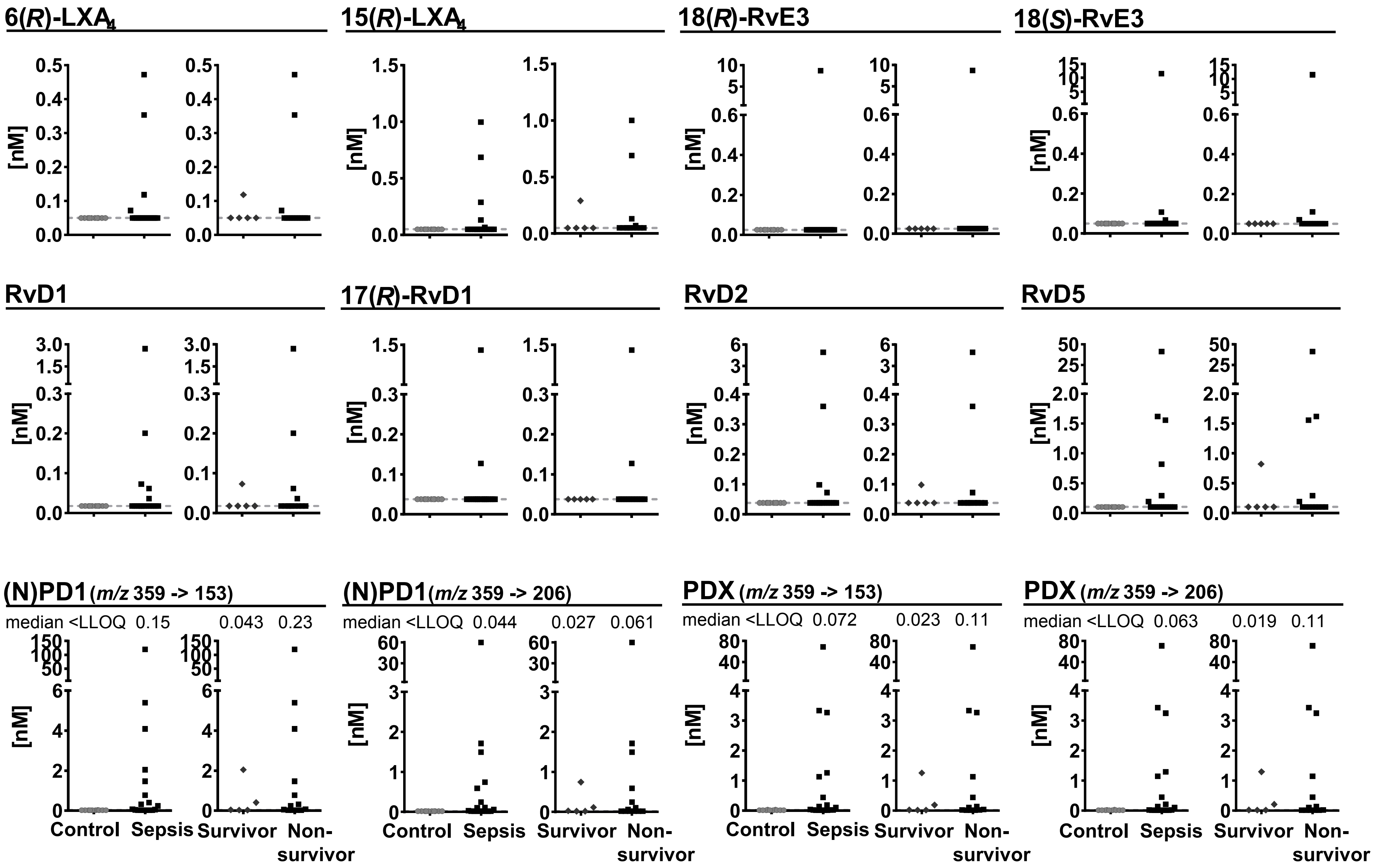

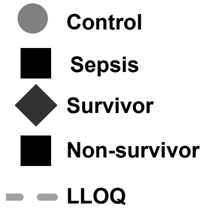

Fig. S8: Concentration of SPMs measured in plasma from patients with (sepsis, n=18) and without (control, n=10) septic shock. Patients with septic shock are divided into survivors (>28 days, n=5) and non-survivors (n=13). Shown are concentrations in nM as individual values of SPMs derived from ARA, EPA and DHA that were not displayed in figure 8. Median is given, if >50% of the samples are >LLOQ. The LLOQ is indicated as dotted line. For (N)PD1 and PDX two transitions are displayed. PDX shows good agreement between determined concentrations for both transitions, while for (N)PD1 the transitions lead to different apparent concentrations, probably due to matrix interferences.
